# Activation of prefrontal parvalbumin interneurons ameliorates working memory deficit even under clinically comparable antipsychotic treatment in a mouse model of schizophrenia

**DOI:** 10.1038/s41386-023-01769-z

**Published:** 2023-12-04

**Authors:** Yosefu Arime, Yoshito Saitoh, Mikiko Ishikawa, Chikako Kamiyoshihara, Yasuo Uchida, Kazuki Fujii, Keizo Takao, Kazufumi Akiyama, Noriaki Ohkawa

**Affiliations:** 1 Division for Memory and Cognitive Function, Research Center for Advanced Medical Science, Comprehensive Research Facilities for Advanced Medical Science, Dokkyo Medical University, Tochigi, Japan; 2 Department of Biological Psychiatry and Neuroscience, Dokkyo Medical University School of Medicine, Tochigi, Japan; 3 Division of Membrane Transport and Drug Targeting, Graduate School of Pharmaceutical Sciences, Tohoku University, Sendai, Japan; 4 Department of Behavioral Physiology, Faculty of Medicine, University of Toyama, Toyama, Japan; 5 Life Science Research Center, University of Toyama, Toyama, Japan; 6 Research Center for Idling Brain Science, University of Toyama, Toyama, Japan; 7 Kawada Hospital, Okayama, Japan

**Keywords:** Schizophrenia, Working memory

## Abstract

One of the critical unmet medical needs in schizophrenia is the treatment for cognitive deficits. However, the neural circuit mechanisms of them remain unresolved. Previous studies utilizing animal models of schizophrenia did not consider the fact that patients with schizophrenia generally cannot discontinue antipsychotic medication due to the high risk of relapse. Here, we used multi-dimensional approaches, including histological analysis of the prelimbic cortex (PL), LC-MS/MS-based in vivo dopamine D2 receptor occupancy analysis for antipsychotics, in vivo calcium imaging, and behavioral analyses of mice using chemogenetics to investigate neural mechanisms and potential therapeutic strategies for working memory deficit in a chronic phencyclidine (PCP) mouse model of schizophrenia. Chronic PCP administration led to alterations in excitatory and inhibitory synapses, specifically in dendritic spines of pyramidal neurons, vesicular glutamate transporter 1 (VGLUT1) positive terminals, and parvalbumin (PV) positive GABAergic interneurons located in layer 2–3 of the PL. Continuous administration of olanzapine, which achieved a sustained therapeutic window of dopamine D2 receptor occupancy (60–80%) in the striatum, did not ameliorate these synaptic abnormalities and working memory deficit in the chronic PCP-treated mice. We demonstrated that chemogenetic activation of PV neurons in the PL, as confirmed by in vivo calcium imaging, ameliorated working memory deficit in this model even under clinically comparable olanzapine treatment which by itself inhibited only PCP-induced psychomotor hyperactivity. Our study suggests that targeting prefrontal PV neurons could be a promising therapeutic intervention for cognitive deficits in schizophrenia in combination with antipsychotic medication.

## Introduction

One of the critical unmet medical needs in schizophrenia is an effective treatment for cognitive deficits [1]. A therapeutic intervention for deficits in cognitive functions such as working memory and executive function is challenging due to the following reasons: (1) poor clinical [2] and long-term functional outcomes [3]; (2) very high frequency [4]; and (3) commonly stable over life-time [5, 6]. Unfortunately, existing medications, including antipsychotics, have limited efficacy in treating cognitive deficits [7, 8]. Therefore, there is an urgent need for novel pharmacotherapies that can effectively address deficits in patients with schizophrenia. Several meta-analyses suggest that the effects of existing cognitive enhancers on overall cognition and cognitive subdomains are minimal or insignificant [9, 10]. These clinical data highlight the importance of elucidating neural circuit abnormalities that are specific to cognitive deficits in schizophrenia and developing circuit mechanism-based pharmacotherapies for them [11].

To address the above issue, investigating animal models generated by schizophrenia-relevant perturbation will be quite useful. In humans, chronic phencyclidine (PCP) use induces a wide-ranging schizophrenia-like symptomatology, including positive and negative symptoms, and cognitive deficits [12–15]. In rodents, repeated administration of PCP leads to various behavioral phenotypes resembling those of schizophrenia: hypersensitivity to acute PCP treatment; impaired sociability; and deficits in cognitive functions, such as executive function and working memory [16–20]. Hence, chronic PCP-treated rodents have been established as a pharmacological model of schizophrenia [21]. Previously, we demonstrated that the prelimbic cortex (PL) of the medial prefrontal cortex (mPFC), especially layer 2–3, is a candidate brain region responsible for working memory deficit in chronic PCP-treated mice [19]. However, the precise neural circuits that could serve as definitive targets for improving working memory deficits in this mouse model have not yet been elucidated.

Despite significant progress in basic research using animal models, there remain some translational gaps between this research and the clinical reality of schizophrenia. In patients with schizophrenia, antipsychotic medication is crucial not only for improving positive symptoms in the acute phase but also for preventing relapse in the maintenance phase [22]. All current pharmacological treatments for schizophrenia comprise dopamine D2 receptor antagonists. PET studies have suggested that an optimal therapeutic window with antipsychotics can be obtained by 60%–80% occupancy of striatal dopamine D2 receptors [23, 24]. Therefore, improving cognitive deficits in patients with schizophrenia, while simultaneously managing their psychotic symptoms with antipsychotic treatment, is essential. Despite several studies using animal models of schizophrenia, the neurobiological mechanisms underlying cognitive deficits in schizophrenia remain poorly understood and unaddressed, and a potential remedy for them has not been fully explored.

To overcome these challenges and bridge the translational gaps, we aimed to explore a circuit-based therapeutic approach to ameliorate working memory deficit in chronic PCP-treated mice, even under concurrent continuous antipsychotic treatment. We first analyzed synaptic pathology of the PL, associated with working memory deficit in chronic PCP-treated mice [19], focusing on the dendritic spines and excitatory and inhibitory synapses including parvalbumin (PV)-positive interneurons. We proceeded to manipulate the PV interneurons using the chemogenetic technique of designer receptors exclusively activated by designer drugs (DREADDs), which we confirmed through in vivo calcium imaging. Additionally, we observed significant improvement in the working memory deficit of chronic PCP-treated mice following perturbation of the neural circuit in the PL. Furthermore, we established an antipsychotic dose regimen that could maintain clinically relevant levels of striatal dopamine D2 receptor blockade in mice for multiple weeks using LC-MS/MS-based in vivo receptor occupancy analysis and assessed whether the effects of these dose regimen on histological and behavioral abnormalities. Finally, we analyzed whether specific PV activation in the PL ameliorates working memory deficit in this mouse model, even with clinically comparable antipsychotic treatment.

## Materials And methods

For detailed methods and materials, see the Supplementary Information.

### Animals

All animal experiments were performed using the protocol approved by the Ethics Committee of Dokkyo Medical University (Permit Number: 0704, 0771, and 1112) and University of Toyama (A2019OPR-1, and A2019OPR-2), according to the Guidelines for Care and Use of Laboratory Animals, and conformed to all Japanese federal animal welfare rules and guidelines. Male Thy1-GFP line O, PV-Cre (C57BL/6 J background), and C57BL/6 J mice were used (see details in the Supplementary Information). PCP administration was started when mice were 9–10 weeks old for working memory task and dendritic spine and synapse analysis and 6–7 weeks old for open field test.

### Dendritic spine and synapse analysis

The histology, image acquisition, and analysis methods are described in the Supplementary Information.

### Surgery

Detailed information on viral injection, continuous drug administration, and in vivo calcium imaging is provided in the Supplementary Information.

### Chemogenetics

The chemogenetic procedures using PV-Cre mice, hM3D(Gq)-DREADD, and its selective ligand descloroclozapine (DCZ) are described in the Supplementary Information.

### In vivo calcium imaging

The details on in vivo calcium imaging are provided in the Supplementary Information.

### Behavioral tests

The delayed nonmatching-to-position (DNMTP) task with T-maze [19] and open field test [25] were used with slight modifications. See details in the Supplementary Information.

### c-Fos immunohistochemistry

The details on c-Fos immunohistochemistry are described in the Supplementary Information.

### LC-MS/MS-based in vivo dopamine D2 receptor occupancy with continuous antipsychotic treatment

The details on in vivo dopamine D2 receptor occupancy analysis are described in the Supplementary Information.

## Results

### Chronic PCP administration led to synaptic abnormalities in the PL

**Fig. 1:**
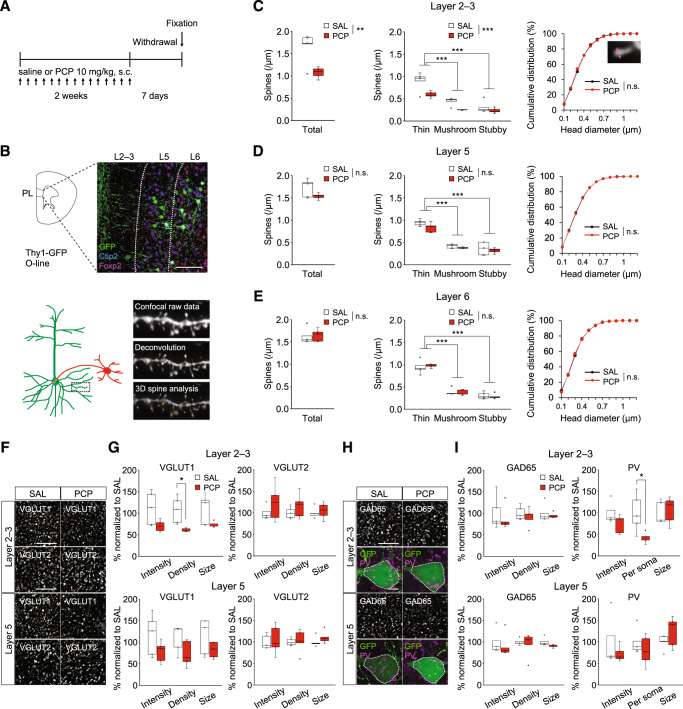
Selective loss of dendritic spines and abnormalities of excitatory and inhibitory synapses in layer 2–3 of the PL of chronic PCP-treated mice. **A** Schematic timeline for morphological analysis. **B** Representative confocal images in the PL of Thy1-GFP line O mice before and after 3D deconvolution, and 3D reconstructions of dendritic spines by NeuronStudio. Scale bar: 100 µm. **C**–**E** Dendritic spine analysis in the PL. In layer 2–3 of the PL (**C**), chronic PCP administration results in decreases in total (left) and each spine density (middle). In layer 5 (**D**) and 6 (**E**), no changes were observed in total (left) and each spine density (middle). Cumulative frequency distributions of overall spine head diameter in layer 2–3 (**C**, right), 5 (**D**, right), and 6 (**E**, right) are plotted. There are no changes in the distribution of spine head size in all layers. **F** Representative images of immunostaining for VGLUT1 and VGLUT2 in the PL of chronic saline- or PCP-treated mice. Scale bars: 5 µm. **G** Quantification of VGLUT1 and VGLUT2 positive puncta. Decreased density of VGLUT1 positive puncta in layer 2–3 of the PL in chronic PCP-treated Thy1-GFP mice were observed (top, left). **H** Representative images of immunostaining for GAD65 and PV in the PL of chronic saline- or PCP-treated Thy1-GFP mice. Scale bars: 5 µm. **I** Quantification of GAD65 and PV positive puncta. Decreased PV positive puncta surrounding pyramidal neuron soma in layer 2–3 of the PL in chronic PCP-treated Thy1-GFP mice were observed (top, right). In box plots, the central mark represents the median and the bottom and top edges of the box indicate the 25th and 75th percentiles, respectively. n.s., no significance. *p < 0.05, **p < 0.01, ***p < 0.001.

First, we investigated the pathological basis for working memory deficit in the PL of chronic PCP-treated mice. To investigate the effect of chronic PCP treatment on spine pathology, we reconstructed three-dimensional dendritic segments and then conducted a dendritic spine analysis with NeuronStudio [26]. The laminar cytoarchitecture in the PL was identified by Ctip2 (a layer 5 marker) and Foxp2 (a layer 6 marker) immunostaining (Fig. 1B). We observed that chronic PCP administration caused decreases in total (Fig. 1C, left) and each type of spine density (Fig. 1C, middle) on pyramidal neurons in layer 2–3 of the PL. Analysis of frequency distribution of head diameter of dendritic spines (Fig. 1C, right), head diameter (Supplementary Fig. S1A) and spine length of each spine type (Supplementary Fig. S1B) revealed no changes in spine proportions, implying that chronic PCP reduced dendritic spine density regardless of spine types in this region. Both in layer 5 and 6, no differences were observed in spine density (layer 5: Fig. 1D left and middle, layer 6: Fig. 1E left and middle) and head diameter (layer 5: Fig. 1D right, layer 6: Fig. 1E right, Supplementary Fig. S1C–F) between chronic saline- and PCP-treated mice. The detailed- and layer specific analysis of dendritic spines presented in this study supports and expands upon findings from previous research [27]. Overall, these results suggest that chronic PCP-treated mice experience abnormalities in receiving excitatory synaptic inputs to layer 2–3 pyramidal neurons of the PL.

Next, we tested whether chronic PCP could affect excitatory and inhibitory pre-synapses in the PL. Neurons in the cerebral cortex receive two primary types of excitatory inputs. Vesicular glutamate transporter 1 (VGLUT1) is predominantly localized within the terminals of cortical projections, while VGLUT2 is localized within the terminals of the thalamus and hypothalamus [28, 29]. We observed that chronic PCP administration led to a decrease in VGLUT1+ puncta density in layer 2–3 of the PL with no change in layer 5 (Fig. 1G, left). Both in layer 2–3 and layer 5, no differences were observed in VGLUT2+ puncta between chronic saline- and PCP-treated mice. (Fig. 1G, right). Together, our results demonstrate that cortical excitatory inputs to layer 2–3 of the PL were specifically attenuated in this PCP model. Previous studies reported that subchronic NMDA receptor antagonists lowered PV neuron density in the mPFC [20, 30]. To investigate whether inhibitory synaptic inputs in the PL are influenced by chronic PCP administration, we evaluated inhibitory synapses by quantifying puncta of glutamic acid decarboxylase 65 (GAD65), as a marker of GABAergic neuron terminals, and PV immunoreactive puncta surrounding pyramidal neuron soma, presumed PV+ basket cell terminals [31]. We found that chronic PCP administration caused decrease in PV+ puncta surrounding GFP+ pyramidal neuron cell bodies in layer 2–3 of the PL (Fig. 1I, right). Both in layer 2–3 and layer 5, no differences were observed in GAD65+ puncta between chronic saline- and PCP-treated mice. (Fig. 1I, left). Overall, these anatomical data indicated that PV+ puncta density onto pyramidal neuron soma was lowered in the layer 2–3 of the PL, suggesting attenuated inhibitory inputs from PV+ basket cells in chronic PCP-treated mice.

### Chemogenetic activation of prefrontal PV neurons restores working memory deficit in chronic PCP-treated mice

**Fig. 2:**
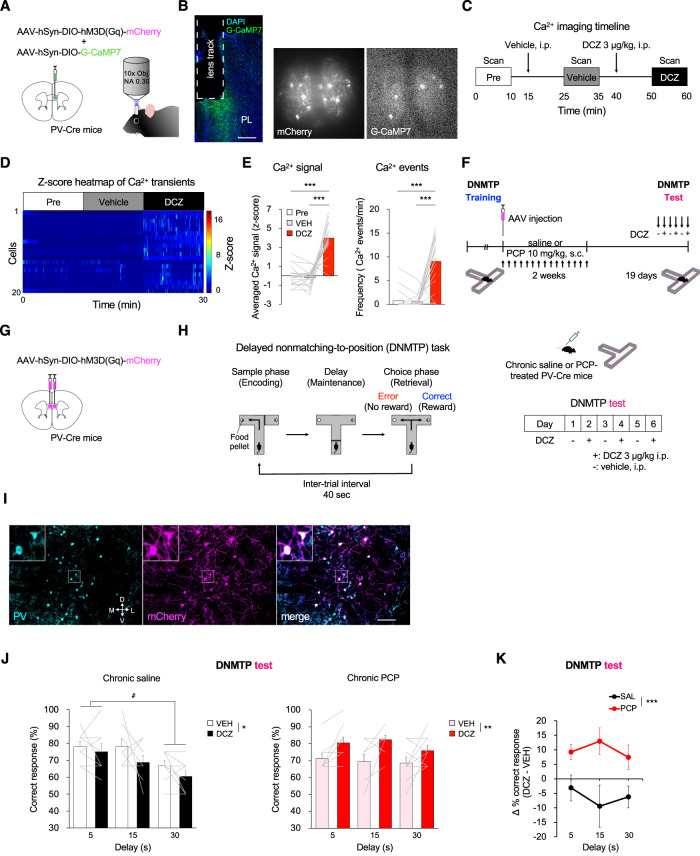
Chemogenetic activation of PV neurons in the PL improves working memory deficit in chronic PCP-treated mice. **A** Schema of viral delivery of AAV-hSyn-DIO-G-CaMP7 and AAV-hSyn-DIO-hM3D(Gq)-mCherry to PL (left). Schematic diagram of PV neuron activity imaging in the PL of an awake mouse (right). **B** GRIN lens implantation (dashed line) into the PL. Scale bar: 300 µm (left). Representative mCherry (middle) and stacked G-CaMP7 (right) images acquired through Nipkow-disk confocal microscopy. **C** Schematic timeline of confocal scanning. **D** *Z*-score heatmap of average Ca^2+^ transients during pretreatment, vehicle and DCZ 3 µg/kg treatment. **E** Comparison of *z*-score of average Ca^2+^ signals (left) and Ca^2+^ event frequency (right) in 10 min bins. DCZ 3 µg/kg treatment significantly increased both averaged Ca^2+^ signals and frequency of Ca^2+^ event of PV neuron. Data are presented as the mean. Each line represents each cell. ***p < 0.001. **F** Schematic timeline of DNMTP test with T-maze. **G** Schema of bilateral viral delivery of AAV-hSyn-DIO-hM3D(Gq)-mCherry to the PL. **H** Schematic illustration of test sessions conducted in DNMTP task with T-maze (left). Test sessions of DNMTP task consist of six days with DCZ 3 µg/kg every other day (right). **I** Representative images of PV immunostaining (cyan) and viral expression (magenta) in the PL. Scale bar: 100 µm. **J** DCZ 3 µg/kg significantly decreased the percentage of correct responses in chronic saline-treated PV-Cre mice (left). DCZ 3 µg/kg significantly increased the percentage of correct responses across all delay periods in chronic PCP-treated PV-Cre mice that previously received AAV-hSyn-DIO-hM3D(Gq)-mCherry (right). Data are presented as the mean ± SEM. Each line represents each mouse. *p < 0.05, **p < 0.01, ^#^*p* < 0.05. **K** Difference in percent correct responses between chronic saline- and PCP-treated PV-Cre mice. Line graph shows mean ± SEM. ***p < 0.001.

Because our histological data demonstrate that chronic PCP-treated mice exhibited deficits in PV neuron-mediated excitatory and inhibitory circuits in the PL, we subsequently examined whether prefrontal PV neuron activation could restore working memory deficit in this mouse model. We first administered hM3D(Gq), the Gq-coupled excitatory DREADD, and G-CaMP7 to PV interneurons via adeno-associated viruses (AAV)-hSyn-DIO-hM3D(Gq)-mCherry and AAV-hSyn-DIO-G-CaMP7 coinjection into the PL of PV-Cre mice (Fig. 2A, left). After implantation of a gradient refractive index (GRIN) lens in the PL, we imaged the calcium signals of PV neuron during pretreatment, vehicle and deschloroclozapine (DCZ) treatment using Nipkow-disk confocal microscopy (Fig. 2A, right and 2B). Chemogenetic perturbation has been used as a powerful tool for manipulating neuronal activity and behaviors. Recent research has indicated that clozapine-N-oxide (CNO), a widely used DREADD agonist, does not penetrate the brain following systemic administration, and rapidly transformed to clozapine, which readily enters the brain, and activates DREADDs, thus potentially causing unintended effects [32]. In contrast, a novel DREADD actuator DCZ, displays remarkable potency, high brain penetrance, metabolic stability, and selectivity for hM3D(Gq) and hM4D(Gi) DREADDs in mice [33]. To minimize off-target effects and to enhance specificity of PV neurons, we used a new approach for increasing prelimbic PV neuron activity using an excitatory DREADD and its ligand DCZ. We confirmed increased Ca^2+^ signal intensities and frequency of Ca^2+^ events of PV neurons after systemic DCZ 3 µg/kg treatment, which suggests that prelimbic PV neuron activities were elevated by hM3D(Gq)-DREADD (Fig. 2C–E, Supplementary Fig. S2). To determine whether prelimbic PV neuron activation ameliorate working memory deficit, PV-Cre mice that were injected with AAV-hSyn-DIO-hM3D(Gq)-mCherry into the bilateral PL were tested after the completion of chronic saline or PCP treatment (Fig. 2F–I). Similar to the previous studies [30, 34, 35], we observed that prefrontal PV activation significantly reduced working memory in chronic saline-treated PV-Cre mice (Fig. 2J, left). In contrast, compared with vehicle injection, DCZ 3 µg/kg caused a significant increase in percentage of correct responses across all delay periods in chronic PCP-treated PV-Cre mice that previously received AAV-hSyn-DIO-hM3D(Gq)-mCherry (Fig. 2J, right). Comparisons of the two mouse groups (saline vs PCP) revealed that the changes in correct responses by DCZ administration in PCP group increased significantly compared with those in saline group (Fig. 2K). The response latencies to reach pellet cup stayed the same regardless of DCZ treatment (Supplementary Fig. S3A) or behavioral responses (Supplementary Fig. S3B), suggesting that improved performance reflected improved working memory. These results indicate that chemogenetic activation of prelimbic PV neurons improves working memory deficit in chronic PCP-treated mice.

### LC-MS/MS-based in vivo dopamine D2 receptor occupancy with continuous antipsychotic treatment

**Fig. 3:**
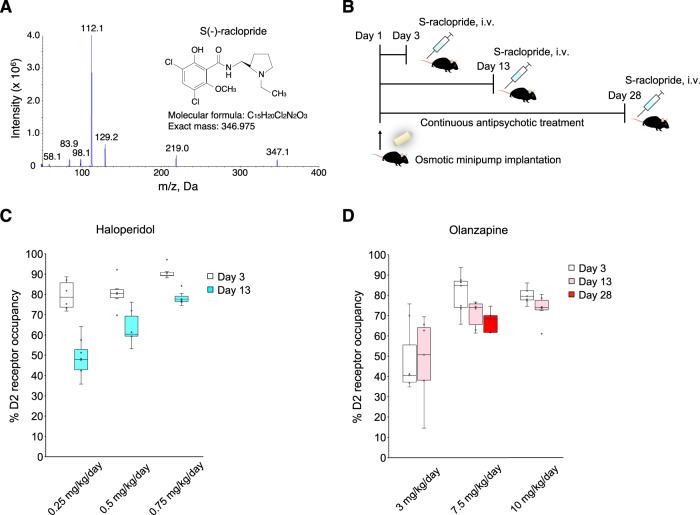
In vivo striatal dopamine D2 receptor occupancy during continuous antipsychotic treatment in mice. **A** Fragmentation pattern of S(-)-raclopride during product ion scan. **B** Schematic timeline of continuous antipsychotic treatment with osmotic minipump. **C** D2 receptor occupancy levels produced by haloperidol, a typical antipsychotic drug. **D** D2 receptor occupancy levels produced by olanzapine, an atypical antipsychotic drug. Clinical levels of dopamine D2 receptor occupancy (60–80%) were reached with haloperidol 0.5 mg/kg/day for 2 weeks and olanzapine 7.5 mg/kg/day for 4 weeks. In box plots, the central mark represents the median and the bottom and top edges of the box indicate the 25th and 75th percentiles, respectively.

We investigated whether D2 receptor occupancy (60–80%) could be achieved and sustained through continuous treatment with haloperidol (typical antipsychotic drug) and olanzapine (atypical antipsychotic drug), which is an indicator of the clinical situation for several weeks, in mice. The objective of this experiment is twofold: (1) to establish antipsychotic doses that could maintain clinically relevant levels of striatal D2 receptor blockade in mice for several weeks, and (2) apply the doses as a tool to validate their ability for synaptic and behavioral abnormalities in the PCP model. We performed in vivo receptor occupancy assays in the mouse striatum for dopamine D2 receptors (Fig. 3A, details see in Supplemental Methods and Supplementary Table S2–4). We continuously administered haloperidol or olanzapine to mice for a maximum of 4 weeks (Fig. 3B). In vivo D2 receptor occupancy analysis revealed that haloperidol 0.5 mg/kg/day achieved and sustained clinical levels of dopamine D2 receptor occupancy (~60–80%) for at least 13 days (Fig. 3C) and olanzapine 7.5 mg/kg/day maintained those levels for at least 28 days (Fig. 3D, Supplementary Table S5).

### Effects of clinically relevant antipsychotic dose regimen on histological and behavioral abnormalities in chronic PCP-treated mice

Current antipsychotic drugs are relatively effective for positive symptoms but have minimal or no effects on cognitive deficits [1, 22]. Based on these clinical situations, we hypothesized that continuous antipsychotic treatment, which maintains clinical levels of dopamine D2 receptor occupancy in mouse striatum, could alleviate acute PCP-induced psychomotor behavioral symptoms, but may fail to improve working memory deficit in chronic PCP-treated mice.

**Fig. 4:**
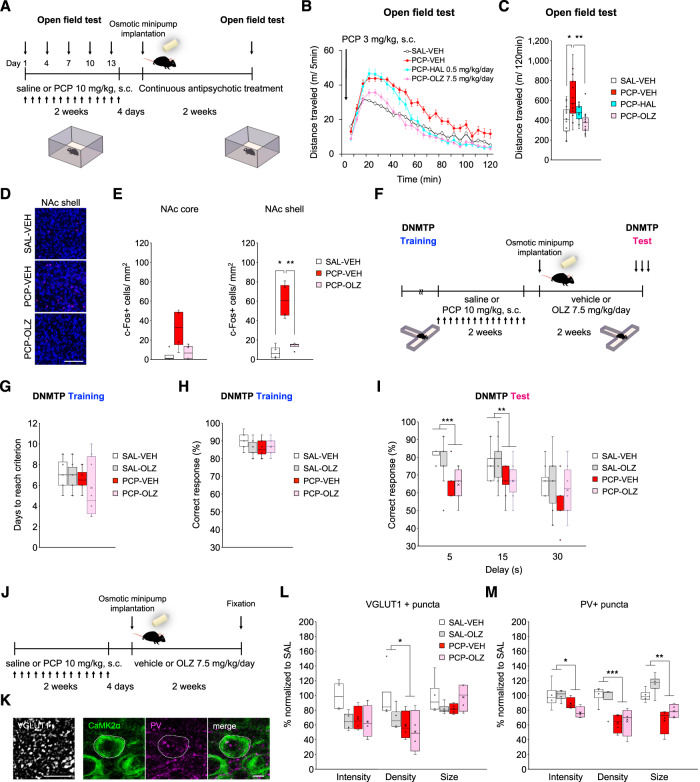
Continuous olanzapine treatment does not ameliorate synaptic abnormalities of the PL and working memory deficit in chronic PCP-treated mice. **A** Schematic timeline of open field test procedure. **B** Distance traveled in 5 min bins following acute PCP 3 mg/kg challenge in the open field during continuous antipsychotic treatment. **C** Acute PCP treatment significantly increased locomotor activity in chronic PCP-treated mice. Compared with continuous vehicle treatment, olanzapine (OLZ) 7.5 mg/kg/day significantly reduced acute PCP-induced locomotor hyperactivity in chronic PCP-treated mice. **D** Representative images of c-Fos immunostaining in the shell of the nucleus accumbens (NAc). Scale bar: 100 µm. **E** Quantification of c-Fos positive cells in the NAc core (left) and shell (right). Acute PCP treatment significantly increased c-Fos positive cells in the NAc shell of chronic PCP-treated mice. Compared with continuous vehicle treatment, OLZ 7.5 mg/kg/day significantly reduced acute PCP-induced c-Fos elevation in chronic PCP-treated mice. **F** Schematic timeline of DNMTP task with T-maze with continuous olanzapine treatment. During training sessions of DNMTP task, no differences were observed in the number of days taken to reach criterion (**G**) and correct responses (**H**) were observed before repeated administration of saline or PCP. **I** Similar to our previous study, repeated administration of PCP reduced percentage of correct responses during DNMTP task with T-maze. OLZ 7.5 mg/kg/day (PCP-OLZ 7.5 mg/kg/day) did not affect working memory in either chronic saline- or PCP-treated mice. **J** Schematic timeline of morphological analysis after continuous vehicle or OLZ treatment both in chronic saline- or PCP-treated mice. **K** Representative images of immunostaining for VGLUT1 (left), CaMK2a (right, green), and PV (right, magenta). Scale bars: 5 µm. **L** Quantification of VGLUT1 positive puncta. Decreased density of VGLUT1 positive puncta in layer 2–3 of the PL in chronic PCP-treated mice were observed. OLZ 7.5 mg/kg/day for two weeks did not affect VGLUT1 positive puncta in layer 2–3 of the PL either in chronic saline- or PCP-treated mice. **M** Quantification of PV positive puncta surrounding CaMK2a positive pyramidal neuron soma. Decreased expression of PV (intensity, per soma, and size) in layer 2–3 of the PL in chronic PCP-treated mice was observed. OLZ 7.5 mg/kg/day for two weeks did not affect PV positive puncta in layer 2–3 of the PL both in chronic saline- and PCP-treated mice. In box plots, the central mark represents the median and the bottom and top edges of the box indicate the 25th and 75th percentiles, respectively. n.s., no significance. *p < 0.05, **p < 0.01, ***p < 0.001.

To evaluate this, we initially assessed whether the maintenance of clinical levels of dopamine D2 receptor occupancy for at least 2 weeks via continuous treatment with haloperidol or olanzapine would affect acute PCP challenge induced hyperlocomotion (Fig. 4A). The open field test revealed that repeated PCP administration induced behavioral sensitization across all groups (Supplementary Fig. S4A–F). After withdrawal from PCP treatment, acute PCP-induced locomotor activity and c-Fos expression in the nucleus accumbens (NAc) were measured. Acute PCP 3 mg/kg challenge induced hyperlocomotion in mice chronically treated with PCP (Fig. 4B, C) and increased the number of entries and distance traveled both in the corner and center zones (Supplementary Fig. S5A–D). Only olanzapine 7.5 mg/kg/day attenuated acute PCP-induced hyperactivity (Fig. 4C) and reduced the number of entries and distance traveled both in the corner and center zones (Supplementary Fig. S5A–D). Acute PCP challenge with open field test also increased c-Fos expression in the shell of the NAc (Fig. 4D, E). Olanzapine 7.5 mg/kg/day reduced acute PCP-induced c-Fos elevation in this region.

Next, we analyzed whether continuous olanzapine treatment would influence working memory deficit in chronic PCP-treated mice (Fig. 4F). Working memory was evaluated using delayed nonmatching-to-position (DNMTP) task with T-maze which has been described previously [19]. During DNMTP training, we observed no significant model × treatment interaction and main effects of them in either the number of days taken to meet the criterion (Fig. 4G) or in correct response rates (Fig. 4H) prior to continuous vehicle or olanzapine treatment. Consistent with our previous investigation [19], repeated administration of PCP resulted in a reduction in the percentage of correct responses during the DNMTP task with T-maze (Fig. 4I). We then found that administration of olanzapine 7.5 mg/kg/day had no effects on the percentage of correct responses in both chronic saline- and PCP-treated mice. These results indicated that clinically comparable olanzapine treatment ameliorates psychomotor behavior and neural hyperactivation in the shell of the NAc but does not affect working memory deficit in our PCP model, suggesting that they share similar poor clinical efficacy with antipsychotics.

We then tested whether continuous olanzapine treatment could affect synaptic abnormalities in layer 2–3 of the PL of chronic PCP-treated mice (Fig. 4J). We found that repeated administration with PCP reduced VGLUT1+ (Fig. 4L) and PV+ puncta (Fig. 4M) and that continuous treatment with olanzapine 7.5 mg/kg/day had no effect on these synaptic abnormalities both in chronic saline- and PCP-treated mice. Previous studies have demonstrated that prefrontal PV+ neuron density was reduced in rodents chronically treated with NMDA receptor antagonists [20, 30] and in patients with schizophrenia [36]. Similar to these findings, we observed that repeated administration of PCP reduced PV+ neuron density in the PL approximately three weeks after withdrawal from PCP (Supplementary Fig. S6A, B). We also found olanzapine 7.5 mg/kg/day had no effect on reduced PV+ neuron density. Taken together, our findings indicate the presence of persistent abnormalities in prelimbic PV neurons of mice subjected to chronic PCP treatment, and that continuous administration of olanzapine does not affect the excitatory and inhibitory synaptic abnormalities in the PL, especially in layer 2–3, as well as working memory deficit in our PCP model.

### Chemogenetic activation of prefrontal PV neurons combined with clinically comparable olanzapine treatment restores working memory deficit in chronic PCP-treated mice

**Fig. 5:**
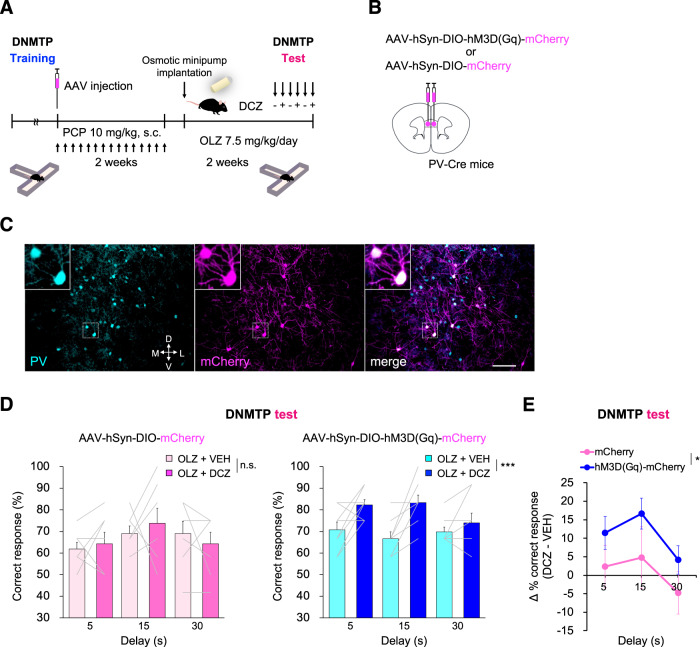
Chemogenetic activation of PV neurons in the PL improves working memory deficit in chronic PCP-treated mice even under continuous OLZ treatment. **A** Schematic timeline of DNMTP task with T-maze. **B** Schema of bilateral viral delivery of AAV-hSyn-DIO-mCherry or AAV-hSyn-DIO-hM3D(Gq)-mCherry to the PL. **C** Representative images of PV immunostaining (cyan) and viral expression (magenta) in the PL. Scale bar: 100 µm. **D** The percentage of correct responses was not changed by DCZ in chronic PCP-treated PV-Cre mice that previously received AAV-hSyn-DIO-mCherry during continuous OLZ treatment (left). DCZ 3 µg/kg significantly increased the percentage of correct responses across all delay periods in chronic PCP-treated PV-Cre mice that previously received AAV-hSyn-DIO-hM3D(Gq)-mCherry during continuous OLZ treatment (right). Data are presented as the mean ± SEM. Each line represents each mouse. n.s., no significance. **p < 0.01. **E** Difference in percent correct responses between DCZ and vehicle treatment in AAV-hSyn-DIO-mCherry- and AAV-hSyn-DIO-hM3D(Gq)-mCherry-injected mice. Line graph shows mean ± SEM. *p < 0.05.

We investigated whether the ameliorative effect of prefrontal PV activation via hM3D(Gq)-DREADD on working memory persists even under treatment with clinically relevant antipsychotic doses, which reflect the medication status of patients with schizophrenia. We administered hM3D(Gq)-DREADD to PV interneurons by AAV-hSyn-DIO-hM3D(Gq)-mCherry injection into the PL (Fig. 5A, B). AAV-hSyn-DIO-mCherry was used to control potential effects of DCZ. We virally targeted hM3D(Gq)-mCherry or mCherry to PV neurons in the PL of chronic PCP-treated PV-Cre mice with subsequent olanzapine 7.5 mg/kg/day treatment (Fig. 5C). In DNMTP training, no significant differences were observed in the number of days taken to meet the criterion (Supplementary Fig. S7A) or in correct response rates (Supplementary Fig. S7B) between the two types of AAV groups noted above. In DNMTP test sessions with olanzapine 7.5 mg/kg/day treatment, we observed that DCZ 3 µg/kg led to a significant increase in percentage of correct responses across all delay periods in chronic PCP-treated PV-Cre mice that previously received AAV-hSyn-DIO-hM3D(Gq)-mCherry (Fig. 5D, right). Such a recovery effect was not observed in chronic PCP-treated PV-Cre mice that previously received AAV-hSyn-DIO-mCherry (Fig. 5D, left). Comparisons of the two mouse groups (mCherry vs hM3D(Gq)-mCherry) revealed that the changes in correct responses by DCZ administration in hM3D(Gq) group increased significantly compared with those in mCherry group (Fig. 5E). Changes in correct response rate were not observed between mCherry- and hM3D(Gq)-expressing mice under the absence of DCZ (Supplementary Fig. S9). Response latency did not change according to treatment conditions (Supplementary Fig. S8A) or behavioral responses (Supplementary Fig. S8B), suggesting that improved performance reflects improved working memory. To further examine the impact of prefrontal PV activation on behavior in PCP model, we performed the open field test in chronic PCP-treated PV-Cre mice that previously received AAV-hSyn-DIO-mCherry or AAV-hSyn-DIO-hM3D(Gq)-mCherry under olanzapine 7.5 mg/kg/day treatment. We found that DCZ 3 µg/kg did not alter acute PCP-induced locomotor activity between hM3D(Gq)-mCherry- and mCherry-expressing mice, suggesting that prefrontal PV activation does not affect inhibitory effect of continuous olanzapine treatment on psychomotor behavior (Supplementary Fig. S10D, E). Overall, these results demonstrate that chemogenetic activation of prelimbic PV neurons ameliorate working memory deficit in chronic PCP-treated mice, even under continuous olanzapine treatment without influencing improvement effects of olanzapine on psychomotor hyperactivity.

## Discussion

Our study revealed that chronic PCP administration leads to working memory deficit, decreased excitatory inputs from other cortical regions, and a reduction in the PV neuron terminals onto the pyramidal neuron soma in layer 2–3 of the PL. None of these effects were rescued by chronic antipsychotic treatment. However, chemogenetic activation of PV neurons in this region improved working memory deficit in a PCP mouse model, even under continuous olanzapine treatment. In contrast, prefrontal PV activation did not affect the therapeutic effects of continuous olanzapine on acute PCP-induced hyperactivity. Our findings using this pharmacological model of schizophrenia support the hypothesis that an abnormal balance between excitatory and inhibitory tones in the PFC plays a key role in the pathophysiology of cognitive deficits observed in schizophrenia [37, 38] and the results presented here suggest that prefrontal PV neuron activation may offer a promising approach to addressing working memory deficit in patients with schizophrenia who require continuing antipsychotic medication.

Various cognitive tasks, such as the novel object recognition task and other similar tasks, have been commonly used in preclinical studies. While current antipsychotics have demonstrated minimal or even adverse effects on cognitive deficits in clinical settings, several preclinical studies have indicated that these same drugs can improve deficits in various cognitive tasks. However, the inconsistent results from preclinical and clinical studies suggest limited predictive validity [39]. We found that continuous olanzapine treatment, which sustains clinical levels of dopamine D2 receptor occupancy in the mouse striatum for at least 4 weeks, could ameliorate acute PCP-induced psychomotor behavior and c-Fos elevation in the shell of the NAc, suggesting as the primary site of action of antipsychotics on hyperlocomotion, but not improve working memory deficit in chronic PCP-treated mice. These findings suggest that our PCP model accurately represents cognitive deficits that remain unchanged when treated with antipsychotics. The presence of enduring working memory deficits against antipsychotic treatment in our PCP model suggests that it could be a useful tool to explore novel therapeutic strategies for cognitive deficits in schizophrenia.

To develop potential therapeutic strategies for cognitive deficits in schizophrenia, it is essential to verify the neural substrates across a range of scales from molecular and cellular to behavioral levels in preclinical models relevant to schizophrenia [40]. There is growing evidence to suggest that the balance between excitatory and inhibitory network in the mPFC is crucial for the regulation of working memory in normal mice [34, 35, 41, 42]. Decreased dendritic spine density in the dorsolateral prefrontal cortex (DLPFC) of patients with schizophrenia has been replicated in numerous studies [43–45] and proposed to play a key pathophysiological role in schizophrenia [46]. The dendritic spine analysis presented here clearly indicated that chronic PCP administration leads to dendritic spine loss in layer 2–3 of the PL. Elsworth et al. reported dendritic spine loss in the PFC of chronic PCP-treated rats and reversal by chronic oral olanzapine [27]. This discrepancy might be due to experimental design (route of olanzapine, PCP dosage, and sampling time) or origin of dendritic spines (basal dendritic spines of layer 2–3 pyramidal cells vs dendritic spines in layer 2–3). We also observed reduction in VGLUT1+ puncta in this region, suggesting that the density of excitatory inputs in this region was reduced. Previous studies have revealed that the size of dendritic spine head is closely linked to synaptic function such as the size of postsynaptic density, the number of AMPA receptors, and synaptic strength [47–49]. These anatomical changes observed in the present study suggest that our PCP model exhibits attenuated excitatory inputs from other cortex in the PL, which is consistent with the morphological features observed in patients with schizophrenia, such as reductions in dendritic spines [43–45, 50] and VGLUT1 [51, 52] in layer 3 of the DLPFC.

Prefrontal interneuron impairments have been hypothesized to contribute to cognitive deficits in schizophrenia [36, 53]. However, the causal relationship between their abnormalities and cognitive deficits remains unclear. Repeated blockade of NMDA receptors during early adulthood has been reported to reduce the density of PV neurons in the mPFC, and this was attributed to downregulation of PV expression levels rather than neuronal death [54, 55]. The present study revealed a decrease not only in PV density but also in PV+ puncta onto the pyramidal neuron soma, presumed PV+ basket cell terminals, in layer 2–3 of the PL. These results imply that chronic PCP administration during early adulthood leads to hypofunction of prelimbic PV neurons in a layer specific manner, which are in line with post-mortem brain studies of schizophrenia [36, 37]. It is known that disinhibition of pyramidal cells occurs due to suppression of inhibitory neurons, which may be caused by PV neuron defects [56]. Additionally, we previously observed that PCP model showed c-Fos overexpression in layer 2–3 of the PL after working memory load [19]. Based on these results, we hypothesized that attenuated inhibition from PV neurons to excitatory pyramidal neurons and these structural changes, may underlie the potential neurobiological basis for cognitive deficits. Notably, in addition to working memory deficit, these abnormalities in excitatory and inhibitory synapses of the PL in our PCP model were antipsychotic-resistant.

Our histological findings suggest that targeting PV neurons in the PL has the potential to improve working memory deficit in chronic PCP-treated mice. Indeed, previous studies using pharmacological [20] or genetic [57] animal models of schizophrenia have found evidence of prefrontal PV neuron defects at cellular levels, although the causal relationship between their abnormalities and cognitive deficits remains elusive. Recently, Chamberlin et al. reported that activation of PV neurons in the PL using excitatory DREADD and its agonist CNO improved cognitive flexibility in MK-801-treated female rats [30]. In the present study, we found that prelimbic PV activation in vivo alleviates working memory deficit in our PCP model. Although these results do not contradict the recent report [30] and imply that specific increase in prelimbic PV neuron activity is sufficient to improve working memory in different schizophrenia models, our finding has more important advantage that chemogenetic PV activation in this region ameliorated working memory deficit even under continuous olanzapine treatment. In addition, it did not disturb the inhibitory effects of continuous olanzapine treatment on acute PCP-induced hyperactivity, which may be due to sustained 60–80% occupancy of striatal dopamine D2 receptor blockade. Overall, our findings suggest that prefrontal PV activation could be a promising therapeutic strategy to improve cognitive deficits in schizophrenia when used in combination with current antipsychotic medication. On the other hand, our PCP model lacks some construct validity (e.g., genetic, environmental, and developmental factors). Further studies are needed to determine whether prefrontal PV activation could ameliorate cognitive deficits in other animal models of schizophrenia.

This study investigated the neural substrates underlying working memory deficit in PCP model, with the aim of developing therapeutic strategies, by focusing mainly on PV neurons in the mPFC, especially PL. PV+ basket cells targeting the perisomatic pyramidal cells exert a powerful control over their excitability [58, 59]. PL PV+ basket cells differentiate their firing during working memory tasks such that individual cells were recruited or inhibited while rats performed working memory tasks [60], implying discrete contributions of PV+ basket cells in the PL to different task episodes. Moreover, it is known that excitatory drive to PV neurons in all layers of the frontal cortex, including layer 2–3, is primarily VGLUT1+ terminals [61]. Given the results of our puncta analysis, PV neuron deficits in chronic PCP-treated mice may be due to a reduction in VGLUT1+ puncta. A limitation of the present study is that although the chemogenetic approach we used in the present study may have activated individual PV neurons by decreasing their firing threshold in the PL, the underlying circuit mechanisms that mediate this effect remain unclear. PV neurons are essential for the generation of gamma oscillations [62]. Neural oscillations such as gamma band in the mPFC and hippocampus have been linked to spatial working memory in rodents [63]. Gamma oscillations are observed during both sample (encoding) [64] and choice phase (retrieval) [65] of working memory tasks. Patients with schizophrenia exhibit lower amplitudes in gamma band than healthy controls during working memory tasks, and both baseline and working memory-induced gamma show strong dependence on baseline GABA level in the DLPFC [66]. However, temporal involvement of neural oscillations in working memory or its impairment in PCP model remains unclear. Further understanding of the functional dynamics of PV neurons, including neuronal ensemble or oscillatory activity such as gamma band in working memory deficit and any ameliorating effects is required.

In conclusion, our study provides additional evidence to support the causal relationship between prefrontal PV neurons and working memory deficit in schizophrenia. Further to this evidence, we propose that perturbation to prefrontal PV neurons may serve as an add-on therapy to antipsychotics, offering new insights into drug discovery aimed at ameliorating cognitive deficits in schizophrenia.

### Supplementary information

Table S1

Supplemental information
