# Supplementary material for: Activation of prefrontal parvalbumin interneurons ameliorates working memory deficit even under clinically comparable antipsychotic treatment in a mouse model of schizophrenia
Source: Neuropsychopharmacology. 2023 Dec 4;49(4):720–30. doi: 10.1038/s41386-023-01769-z (PMC10876596; doi:10.1038/s41386-023-01769-z)
Supplement: Supplementary file 2 — Supplemental information [file 41386_2023_1769_MOESM2_ESM.pdf]

Activation of prefrontal parvalbumin interneurons ameliorates working memory deficit even under clinically comparable antipsychotic treatment in a mouse model of schizophrenia

Supplementary Information

Supplemental Methods

Supplemental References

Supplemental Figures S1-S10

Supplemental Tables S1-S5

Supplemental Methods

Animals

For dendritic spine and synapses analysis, male mice harboring a green fluorescent protein (GFP) transgene under control of the Thy1 promoter, were specified as Thy1-GFP line O (Strain number: 007919), and were obtained from Jackson Laboratory. Hemizygous male Thy1-GFP line O mice were crossed against inbred female C57BL/6J mice from Japan Clea Co., to give birth to hemizygotes used for breeding and morphological experiments. For in vivo dopamine D2 receptor occupancy analysis, male C57BL/6J mice (8–9 weeks old) were used. Male PV-Cre mice (Strain number: 017320) were used for in vivo Ca^{2+} imaging and DREADD experiments. A total of 2–4 mice were housed per cage in a temperature-controlled (24°C ± 1°C) and light-controlled room (light on from 7 a.m. to 7 p.m.) in plastic cages with *ad libitum* access to food and water, except for food restriction experiments and in vivo Ca^{2+} imaging, was used. PCR-based genotyping for the transgene was conducted with primers and biopsy specimens of mouse tail were collected using Hot Sodium Hydroxide and Tris (HotSHOT) method to extract genomic DNA [1].

Drug

Phencyclidine hydrochloride (P3029, Sigma-Aldrich) was dissolved in saline. The mice were treated with saline or PCP 10 mg/kg each day for 14 consecutive days. Haloperidol (H1512, Sigma-Aldrich) and olanzapine (O1141, Sigma-Aldrich) were dissolved in 2% glacial acetic acid in sterilized water (pH adjusted to ~5 with 1N NaOH). For tail vein administration, S(-)-raclopride (+)-tartrate salt (R121, Sigma-Aldrich), used as an occupancy tracer, was dissolved in saline. For LC-MS/MS analysis, S(-)-raclopride (+)-tartrate salt and (±)-metoprolol (+)-tartrate

salt (M5391, Sigma-Aldrich) were dissolved in 0.1% formic acid (LC-MS grade) in water. Deschloroclozapine (DCZ), is a highly potent and selective DREADD agonist [2], was purchased from MedChemExpress (HY-42110). DCZ was dissolved in 1%–2% dimethyl sulfoxide (DMSO) in saline.

Viral vector preparation

For expressing hM3Dq-mCherry, mCherry, or G-CaMP7 [3] in PV+ neurons, AAV9-hSyn-DIO-hM3D(Gq)-mCherry, AAV9-hSynI-DIO-mCherry, or AAV9-hSynI-DIO-G-CaMP7 was produced as described previously [4,5] with pAAV-hSyn-DIO-hM3D(Gq)-mCherry (Addgene #44361), pAAV-hSynI-DIO-mCherry (Addgene #50459), or pAAV-hSynI-DIO-G-CaMP7 plasmid DNA, respectively. The pAAV-hSynI-DIO-mCherry or the pAAV-hSynI-DIO-G-CaMP7 was constructed by replacing the hM3D(Gq)-mCherry sequence with the mCherry or the G-CaMP7 sequence in the pAAV-hSyn-DIO-hM3D(Gq)-mCherry plasmid using the NheI and AscI sites that were blunted with the Klenow fragment of DNA polymerase I. Viral titer was determined by quantitative polymerase chain reaction.

Surgery and drug treatment

For continuous antipsychotic treatments, osmotic minipump (Alzet, model 1002 for vehicle and haloperidol, or 2002 and 2004 for olanzapine), containing either vehicle (VEH; 2% glacial acetic acid in sterilized water), haloperidol 0.25, 0.5, and 0.75 mg/kg/day or olanzapine 3.0, 7.5, and 10 mg/kg/day, were implanted subcutaneously to provide a steady delivery rate with its flow moderator away from the incision site under avertin or sevoflurane anesthesia. Mice were placed on a heating pad for small animals to maintain their body temperature during surgery. For in vivo

Ca^{2+} imaging and DREADD approaches, surgeries were performed using stereotaxic instruments (SR-5M-HT and SM-15; Narishige). Mice were anesthetized using a mixture of medetomidine, midazolam, and butorphanol.

Immunohistochemical analysis of three-dimensional, detailed, and cortical layer specific dendritic spine analysis in the prelimbic cortex

After 1-week-withdrawal from daily PCP 10 mg/kg or saline administration for 14 days, Thy1-GFP line O mice were anesthetized with pentobarbital (100 mg/kg, i.p.) and xylazine (10 mg/kg, i.p.), transcardially perfused with 1% paraformaldehyde (PFA) in 0.1M phosphate buffer (PB) for 1 min, and then with a fixative containing 4% PFA and 0.125% glutaraldehyde in 0.1M PB. Brains were post-fixed with same fixative for 6 h at 4ºC and then coronally sliced (100 µm thickness) using a slicer (NeoLinear Slicer MT, Dosaka EM, Japan). After washing with Tris-buffered saline (TBS) containing 0.1% Tween 20 (TBST), free-floating sections were incubated with TBS containing 5% normal goat serum (NGS) and 0.3% Triton X-100 for 1h at room temperature (RT), and then for two days overnight at 4ºC in the primary antibody diluted in blocking buffer. We used the following primary antibodies: chicken anti-GFP (1:2000, ab13970, abcam), rat anti-Ctip2 (1:500, ab18465, abcam), rabbit anti-Foxp2 (1:2000, ab16046, abcam). After washing with TBST, sections were incubated for 2 h at RT with goat Alexa 488-conjugated anti-chick IgG (1:500, A11039), goat Alexa 594-conjugated anti-rat IgG (1:500, A11007) and goat Alexa 647-conjugated anti-rabbit IgG (1:500, A21245). After washing, sections were mounted onto glass slides.

Immunohistochemical analysis of excitatory and inhibitory synaptic puncta analysis in the

prelimbic cortex

After 1-week-withdrawal from daily PCP 10 mg/kg or saline administration for 14 days, Thy1-GFP line O mice were anesthetized with sodium pentobarbital (100 mg/kg, i.p.) and xylazine (10 mg/kg, i.p.), and transcardially perfused with 4% PFA and 15% saturation picric acid in 0.1 M PB. For synapse staining, this entire perfusion fixation procedure was quickly performed to prevent the structural changes at synapses [6]. Brains were post-fixed with same fixative for 2 h at 4ºC and transferred to 15% sucrose in 0.1 M PB and then immersed in 30% sucrose in 0.1 M PB for cryoprotection. 20 μm-thick sections were cut with cryostat and stored at -30ºC in a solution containing 30% (v/v) ethylene glycol, 30% (v/v) glycerol and 0.1 M sodium phosphate buffer until use. Free-floating sections were rinsed with TBST, incubated with TBS containing 5% NGS and 0.3% Triton X-100 for 1h at RT and then incubated for two (VGLUT2, GAD65, CaMK2a, and PV) or three (VGLUT1) days overnight at 4ºC with the primary antibodies for synapse staining. We used the following antibodies for synapse puncta analysis: mouse anti-VGLUT1 (1:1000, 135-511, Synaptic Systems), guinea pig anti-VGLUT2 (1:2000, AB2251, Millipore), mouse anti-GAD65 (1:1000, MAB351, Millipore), rat anti-Ctip2 (1:500, ab18465, abcam), mouse anti-CaMK2a (1:2000, 05-532), and mouse anti-parvalbumin (1:2000, P3088, Sigma-Aldrich). After washing with TBST, sections were incubated for 2 h at RT with the secondary antibodies. We used the following secondary antibodies: goat Dylight 405-conjugated anti-rat IgG (1:500, 612-146-120, Rockland), goat Alexa 488-conjugated anti-mouse IgG (1:500, A11029), goat Alexa 594-conjugated anti-mouse IgG (1:500, A11032) and goat Alexa 647-conjugated anti-guinea pig IgG (1:500, A21450). After washing, sections were mounted onto glass slides.

Image acquisition and data analysis for dendritic spines and synapses

Acquisition of fluorescence images and image analyses were performed by a blind experimenter to test conditions. Dendritic spine analysis was performed according to previously described methods [7] with minor modifications. Confocal images were acquired with the LSM780 (Carl Zeiss) with sequential acquisition method using 20 × 0.8 NA objective at 256 × 256 pixels resolution and 63 × 1.4 NA objective at 1024 × 1024 pixels resolution as z-stacks and 2.5× digital zoom. Confocal images were deconvolved with the 3D blind deconvolution software AutoQuant X3 (Media Cybernetics, MD, USA) and spine analysis was performed using the semi-automated software NeuronStudio (http://research.mssm.edu/cnic/tools-ns.html). NeuronStudio classifies spines into three types (thin, mushroom, and stubby) and analyzes dendritic length, spine number, spine head diameter and spine length. For the puncta analysis of VGLUT1, VGLUT2, GAD65 and PV immunostaining, Regions of interest (ROI) of the cortical layer in the prelimbic cortex was determined by ctip2 immunoreactive layer. Confocal images were acquired with a 63 × 1.4 NA objective at 1024 × 1024 pixels resolution and 3× digital zoom. Z-stack images were obtained with approximately 15 μm optical thick sections (e.g., 35 × 0.43 μm optical sections for VGLUT1) excluding section surface and then deconvolved with AutoQuant X3. Quantification of fluorescence intensity, puncta density and particle size in VGLUT1, VGLUT2 and GAD65 were performed using “measure,” “threshold” and “analyze particles” function of ImageJ. Quantification of these in PV was performed using “ROI manager,” “subtract background,” “measure,” “threshold” and “analyze particles” plugins.

In vivo calcium imaging with hM3D(Gq)-DREADD

Mice were anesthetized with a mixture of medetomidine, midazolam, and butorphanol and

placed on a stereotaxic apparatus. The virus solution (200 nL/site) was injected into the left PL (AP: −1.8, ML: 0.3, DV: −2.2 mm from bregma) at a rate of 0.1 μL/min using a borosilicate glass capillary. Adeno-associated viruses (AAVs) AAV9-hSyn-DIO-hM3D(Gq)-mCherry (4×10^{13} vg/mL) and AAV9-hSyn-DIO-G-CaMP7 (4×10^{13} vg/mL) were infused into the target brain areas. The glass capillary was left in place for at least 10 min. After recovery, we implanted a gradient refractive index (GRIN) lens (Inscopix, diameter 0.5 mm; length: 6.1 mm) into the left PL (AP: −1.8, ML: 0.3, DV: −1.9 mm from bregma). We confirmed spherical and chromatic aberrations of GRIN lens using 4.0 μm TetraSpeck Fluorescent Microsphere (Thermo Scientific). The chamber frame was then cemented to fix the mouse head under the microscope. Calcium imaging experiments began at least 4 weeks after the surgery to allow time for optimal viral expression. During imaging, we head-fixed awake mouse on a treadmill. We performed calcium imaging with chemogenetic manipulation by hM3D(Gq)-DREADD. The fluorescence images of mCherry were used as a marker of hM3D(Gq)-expressing PV neurons. Calcium signals from G-CaMP7-expressed PV neurons were imaged for 10 min under each condition (pretreatment, vehicle, and DCZ 3 μg/kg treatment) with 15 min intervals. Vehicle or DCZ was administered for 10 min before imaging. The fluorescence images of G-CaMP7 were acquired at a rate of 7.5 frames/s using a custom spinning disk confocal microscope CSU-X1 (Yokogawa), a sCMOS pco.edge camera (PCO), a 10× 0.3 NA objective (OLYMPUS), 473 nm laser (Lucir), and μManager. The acquired images were first spatially downsampled by a factor of 4. For tracking cells across each condition (pretreatment, vehicle, and DCZ treatment), single session movies were concatenated into one total movie containing all the imaging sessions, followed by smoothing with Gaussian blur filter. ROIs were manually captured using ImageJ Fiji software. Finally, cells were identified using an automated sorting system, HOTARU (High performance

Optimizer to extract spike Timing And cell location from calcium imaging data via lineaR impUlse) [8,9], and the HOTARU output data of Ca^{2+} signals (fluorescence intensity) represent Ca^{2+} activity under each time frame in each identified cell. To determine each cell's pattern of activity across treatment conditions, the data were normalized to baseline levels of each cell (the average of Ca^{2+} signals during pretreatment), binned into 400 ms windows, and the individual z-score was calculated for each cell with the mean and standard deviation in the entire recording duration. We averaged z-scored Ca^{2+} signals for each treatment condition. The Ca^{2+} events were extracted with the thresholds of 3 SD of baseline. The heatmap image of Ca^{2+} activity was created using MATLAB software (Mathworks). After verification that both virus-driven expression and GRIN lens were located in the left PL, imaging data were analyzed.

Delayed nonmatching-to-position (DNMTP) task with T-maze

Working memory was evaluated using delayed nonmatching-to-position task (DNMTP) with T-maze which has been described previously [10], with slight modifications. Male C57BL/6J and PV-Cre mice were 7 weeks old at the beginning of the experiment and maintained at about 85% of free-feeding body weight by supplementary feeding with normal rodent diet (CE-2) in addition to the food pellets delivered in daily sessions. Water was freely available in the home cage. All behavioral analyses were recorded using a CCD camera under approximately 50-lux illumination at the center of the T-maze. Before all experiments, mice were acclimatized to a sound-attenuated room for 30 min. This task was conducted using a T-maze constructed with two side goal arms (30 × 10 × 15 cm). Two sliding guillotine doors were manually operated to keep the test mouse in the starting area (start box) or to block their entry into the left or right goal arm. The DNMTP task in T-maze was performed over four sessions: (1) adaptation, (2) forced-

alternation training, (3) DNMTP training, (4) DNMTP test. In the adaptation procedure, mice were habituated to the T-maze for 2 days. Mice were first placed in the maze with their cagemates and allowed to freely explore all arms for 10 min and then placed in the maze alone for 5 min each once daily. A food pellet (20 mg dustless precision pellets, Bio-Serv), used as reinforcer during tasks, was placed in a pellet cup at the end of each goal arm. In the forced-alternation training, the mouse was forced to alternate between left and right arm to obtain the food reward. The mouse was placed in the start box with either the left and right goal arm open, and then allowed to obtain food pellets. If the mouse did not run after 30 sec, it was gently pushed to initiate movement as appropriate. The guillotine door of the start box was re-opened after a 5 sec delay, and the mouse was allowed to run into the goal arm which was in the opposite direction to that visited before. Subsequently, it was removed from the maze, and put into a holding cage. After a 40 sec inter-trial interval (ITI), the next trial was started. There were 10 trials on each training day, with the direction of the forced run varying in a pseudorandom sequence manner. This forced-alternation training was followed by DNMTP training. The mouse was allowed to enter the opened arm to eat a food pellet, and then was moved back to the start box. After a 5 sec delay, the mouse was allowed to access both goal arms in the choice run. When the mouse selected the goal arm, which was in the opposite direction to that visited before, it was allowed to consume a food pellet. This session was also repeated 10 times in a pseudorandom sequence manner and continued until a criterion of 80% correct responses on three consecutive days was achieved. After reaching the criteria, mice were treated with saline or PCP 10 mg/kg for 14 consecutive days. After a 4-day withdrawal, they were subjected to subsequent experiments. In the DNMTP test sessions, the mouse was subjected to 4 trials after each interval (5, 15, and 30 sec) conducted in a random order at 40 sec ITI. A total of 12 trials for

each delay were conducted over 3 or 6 consecutive days. Percentage of correct responses was calculated as performance index for spatial working memory during training and test sessions. Δ % correct response was calculated as (Correct response rate of DCZ administration) – (Correct response rate of vehicle administration) [11].

After completion of the DNMTP test session, mice were perfused with 4% PFA. Coronal sections of the prelimbic cortex were collected for confirmation of viral transfection when appropriate.

Chemogenetic activation of prefrontal PV neurons during behavioral tests

The procedure is described above in detail. AAV9-hSyn-DIO-mCherry (4×10^{13} vg/mL) or AAV9-hSyn-DIO-hM3D(Gq)-mCherry (4×10^{13} vg/mL) solution (200 nL/site) was injected into the bilateral PL (AP: −1.8, ML: ±0.3, DV: −2.2 mm from bregma). Test sessions of DNMTP task with T-maze and open field test were performed approximately 3–4 weeks after surgery for the experiment by infusing AAVs. DCZ (3 μg/kg) or vehicle was administered intraperitoneally 10–15 min before each behavioral test. After verification that virus-driven expression was located in the bilateral PL, behavioral data were analyzed.

Open field test

All behavioral experiments were recorded with CCD camera and measured by using a video tracking software (ANY-maze; Stoelting Co., USA). The apparatus was illuminated at approximately 300 lux. Before experiments, mice were transferred to acclimatize to the experimental site for 30 min. Mice were given saline or PCP 10 mg/kg s.c., placed individually in the center of grey plastic cages (42 × 42 × 30 cm). Locomotor activity was measured for 120

min every four days (day 1, 4, 7, 10 and 13). To evaluate the effects of continuous antipsychotic treatment with clinically relevant dose regimen on PCP 3 mg/kg s.c. challenge, open field behavior was assessed 12 days after osmotic minipump implantation. The numbers and durations of the following behavioral parameters were recorded: distance traveled; the total time spent in the center zone (24 × 24 cm); the number of entries into the center zone, the total time spent in the four corner zones (9 × 9 cm at each corner); the number of entries into the corner zone.

Immediately after completion of the open field test, mice were perfused with 4% PFA. Coronal sections of the nucleus accumbens, which is an important region of acute PCP-induced locomotor activity and PCP sensitization, were collected for c-Fos immunohistochemistry.

c-Fos immunohistochemistry

Mice were anesthetized with sodium pentobarbital (100 mg/kg, i.p.) and xylazine (10 mg/kg, i.p.), and transcardially perfused with 4% PFA in 0.1 M PB after completion of the open field test. Free-floating sections of the nucleus accumbens were rinsed with TBST, incubated with TBS containing 5% NGS and 0.3% Triton X-100 for 1h at RT and then incubated with rabbit anti-c-Fos antibody (1:500, sc-52, Santa Cruz Biotechnology) overnight at 4ºC. After washing with TBST, sections were incubated with goat Alexa 594-conjugated anti-rabbit IgG (1:500, A11037) for 1 h at RT. The sections were treated with 4',6-diamidino-2-phenylindole (DAPI) (1 μg/ml, Roche Diagnostics, 10236276001) and then washed with TBS. After washing, sections were mounted onto glass slides. Fluorescence images were acquired using a fluorescence microscope (BZ-X710, Keyence). Quantification of the number of c-Fos positive cells in the ROI was conducted as described previously [10].

LC-MS/MS-based in vivo dopamine D2 receptor occupancy analysis with continuous antipsychotic treatment

While the half-life of antipsychotics in humans is usually 12–24 hr, their half-life in rodents tends to be 2–4 hr [12]. Thus, there exists a translational gap between animal models and clinical situations in terms of pharmacokinetic and pharmacodynamic rationale. In vivo binding assays for dopamine D2 receptors in mouse striatum were performed according to a modified method from that published previously [13]. During a direct infusion experiment using triple quadrupole mass spectrometer, the mass spectra for S-raclopride, used as an occupancy tracer, revealed peaks at m/z 347.1. Following optimization of mass spectrometry conditions (Supplementary Table S2), the product ion m/z 112.1 was used for S-raclopride quantification for LC-MS/MS (Figure 3A). Similarly, the product ion m/z 116.1 was used for metoprolol quantification. To optimize a tracer dose, S-raclopride levels were assessed in the striatum and cerebellum after intravenous administration of S-raclopride at doses 3, 10, and 30 μg/kg (Supplementary Table S3). At the 30 min time point, 3 μg/kg showed the highest striatal/cerebellar concentration ratio. To optimize a tracer survival interval between tracer administration and animal sacrifice, S-raclopride levels were assessed at various intervals. The concentration ratio increased with time reaching a maximum at 30 min after S-raclopride administration (Supplementary Table S4). On day 3, 13, and 28 after osmotic minipump implantation, in vivo dopamine D2 receptor occupancy analysis was conducted in continuous vehicle, haloperidol, and olanzapine groups. S-raclopride (occupancy tracer) and metoprolol (internal standard) were quantified using liquid chromatography-tandem mass spectrometry (LC-MS/MS). Individual mice from each group were placed in an acrylic animal holder (φ25 × 101 mm) and administered S-raclopride 3 μg/kg (6 nmol/kg) in sterile saline via their tail vein. Thirty min after S-raclopride injection, mice were

sacrificed by cervical dislocation. The brains were immediately removed, and striatum and cerebellum were rapidly dissected, weighed and stored in 1.5 mL polypropylene centrifuge tubes at −80°C until analysis. The striatal tissue for total binding and the cerebellar tissue for nonspecific binding were used. After brain tissue samples were thawed, four volumes (w/v) of 0.1% formic acid in water were added to the tubes. Tissue samples were homogenized using an ultrasonic homogenizer (Branson, Sonifier 150). For deproteinization in samples, 200 μL of acetonitrile (LC-MS grade), containing 0.1% formic acid, was added to 50 μL of tissue homogenate. The mixture was stirred and centrifuged for 16 min at 16,000 × g at 4ºC. One volume of the supernatant was added to four volumes of 0.1% formic acid in water. Then, one volume of the solution was added to one volume of metoprolol solution, used as an internal standard for LC-MS/MS analysis, and 6.7 μL of the mixture was injected into the LC-MS/MS system. The HPLC-MS/MS analysis was performed by coupling a HPLC system (Eksigent ekspert ultraLC 100-XL; SCIEX) with a triple quadrupole mass spectrometer (QTRAP 5500; SCIEX), equipped with an electrospray ionization probe on the Turbo V source (SCIEX). Chromatographic separation was performed on an ACQUITY UPLC BEH C18 Column (2.1 mm ID × 50 mm, 1.7 μm particles; Waters) at 40ºC. A temperature of 10ºC was maintained in the autosampler throughout experiments. Mobile phase A and B consisted of 0.1% formic acid in water and 0.1% formic acid in acetonitrile, respectively. Compounds were separated and eluted from the column using the following gradient system: 0–2 min 5% B, 2–7 min 5%–100% B, 7–8 min 100%–5% B, 8–18 min 5% B at a flow rate of 0.2 mL/min. After elution from the column, S-raclopride and metoprolol were selectively detected in positive electrospray mode using multiple reaction monitoring (MRM) mode (Supplementary Table S2). To prevent carry-over in LC-MS/MS analysis, 0.1% formic acid in water was injected between analyte injections. To

calculate analyte concentration, the ion counts in the chromatograms were determined by using the quantitation procedures in Analyst software (SCIEX).

Binding potential (BP) of dopamine D2 receptors and receptor occupancy calculations were established for each mouse employing the previously used method [12-14]. The ratio of the striatum minus cerebellum (an index of specific binding)/cerebellum (an index of nonspecific binding) was used to generate an index of the BP. The percent dopamine D2 receptor occupancy by continuous antipsychotic treatment was calculated as follows:

$$\text{D2 receptor occupancy (\%)} = 100 \times \{(BP_{vehicle} - BP_{antipsychotics})/BP_{vehicle}\}$$

$BP_{antipsychotics}$ stands for the average ratio in continuous antipsychotic treatment, and $BP_{vehicle}$ stands for the average ratio in continuous vehicle-treated mice.

Statistical analysis

Statistical analysis was conducted using Excel (Microsoft) and SPSS Statistics software (IBM). Data were analyzed using paired *t*-test, Student's *t*-test, and multiple-group comparisons through one-way ANOVA, two-way ANOVA, two-way repeated measures ANOVA, or three-way repeated measures ANOVA. The Greenhouse-Geisser correction for repeated measures was applied as necessary. Bonferroni or Dunnett T3 *post hoc* test was conducted as appropriate. Cumulative frequency distribution analysis was evaluated using Kolmogorov-Smirnov test. The detailed statistics are indicated in Supplementary Table S1.

Supplemental References

1 Truett GE, Heeger P, Mynatt RL, Truett AA, Walker JA, Warman ML. Preparation of PCR-quality mouse genomic DNA with hot sodium hydroxide and tris (HotSHOT). Biotechniques. 2000;29(1):52, 54.

2 Nagai Y, Miyakawa N, Takuwa H, Hori Y, Oyama K, Ji B, et al. Deschloroclozapine, a potent and selective chemogenetic actuator enables rapid neuronal and behavioral modulations in mice and monkeys. Nat Neurosci. 2020;23(9):1157-67.

3 Ohkura M, Sasaki T, Sadakari J, Gengyo-Ando K, Kagawa-Nagamura Y, Kobayashi C, et al. Genetically encoded green fluorescent Ca2+ indicators with improved detectability for neuronal Ca2+ signals. PLoS One. 2012;7(12):e51286.

4 Suzuki A, Kosugi S, Murayama E, Sasakawa E, Ohkawa N, Konno A, et al. A cortical cell ensemble in the posterior parietal cortex controls past experience-dependent memory updating. Nat Commun. 2022;13(1):41.

5 Konno A, Hirai H. Efficient whole brain transduction by systemic infusion of minimally purified AAV-PHP.eB. J Neurosci Methods. 2020;346:108914.

6 Tao-Cheng JH, Gallant PE, Brightman MW, Dosemeci A, Reese TS. Structural changes at synapses after delayed perfusion fixation in different regions of the mouse brain. J Comp Neurol. 2007;501(5):731-40.

7 Dumitriu D, Rodriguez A, Morrison JH. High-throughput, detailed, cell-specific neuroanatomy of dendritic spines using microinjection and confocal microscopy. Nat Protoc. 2011;6(9):1391-411.

8 Takekawa T, Nomoto M, Asai H, Ohkawa N, Okubo-Suzuki R, Ghandour K, et al. HOTARU: Automatic sorting system for large scale calcium imaging data. bioRxiv. 2022:2022.04.05.487077.

9 Ghandour K, Ohkawa N, Fung CCA, Asai H, Saitoh Y, Takekawa T, et al. Orchestrated ensemble activities constitute a hippocampal memory engram. Nat Commun. 2019;10(1):2637.

10 Arime Y, Akiyama K. Abnormal neural activation patterns underlying working memory impairment in chronic phencyclidine-treated mice. PLoS One. 2017;12(12):e0189287.

11 Moya NA, Yun S, Fleps SW, Martin MM, Nadel JA, Beutler LR, et al. The effect of

selective nigrostriatal dopamine excess on behaviors linked to the cognitive and negative symptoms of schizophrenia. Neuropsychopharmacology : official publication of the American College of Neuropsychopharmacology. 2023;48(4):690-99.

12 Kapur S, VanderSpek SC, Brownlee BA, Nobrega JN. Antipsychotic dosing in preclinical models is often unrepresentative of the clinical condition: a suggested solution based on in vivo occupancy. J Pharmacol Exp Ther. 2003;305(2):625-31.

13 Barth VN, Chernet E, Martin LJ, Need AB, Rash KS, Morin M, et al. Comparison of rat dopamine D2 receptor occupancy for a series of antipsychotic drugs measured using radiolabeled or nonlabeled raclopride tracer. Life Sci. 2006;78(26):3007-12.

14 Samaha AN, Seeman P, Stewart J, Rajabi H, Kapur S. "Breakthrough" dopamine supersensitivity during ongoing antipsychotic treatment leads to treatment failure over time. J Neurosci. 2007;27(11):2979-86.

Supplemental Figures

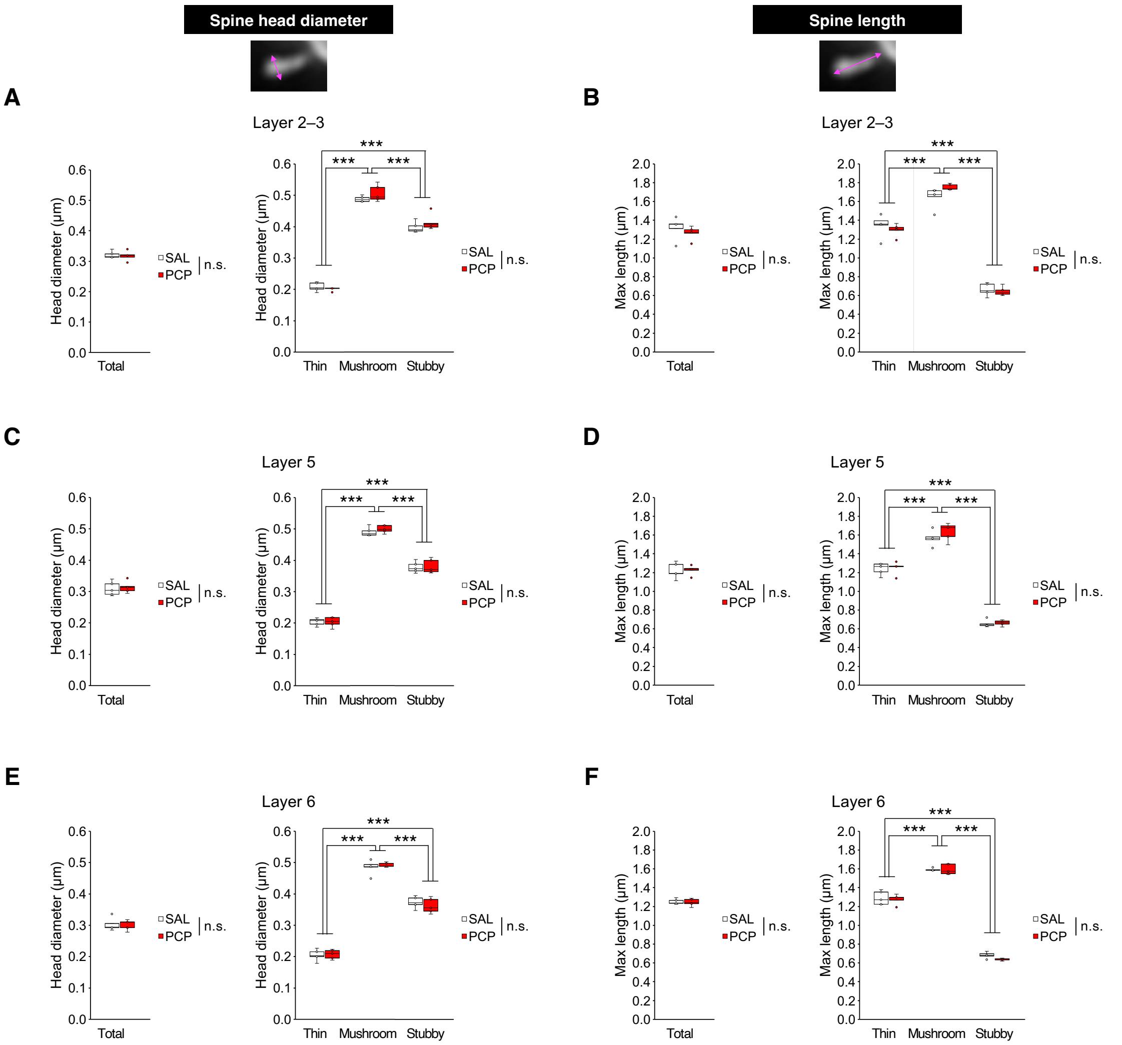

Fig. S1. Head diameter and length of dendritic spines in the PL, related to Fig. 1. (A, C, E) Spine head diameter in the layer 2–3 **(A)**, layer 5 **(C)**, and layer 6 **(E)**. In all layers, no significant differences in head diameter of total and each spine type were observed between groups. **(B, D, F)** Spine length in the layer 2–3 **(B)**, layer 5 **(D)**, and layer 6 **(F)**. In all layers, no significant differences in spine length of total and each spine type were observed between groups. In box plots, the central mark indicates the median and the bottom and top edges of the box indicate the 25th and 75th percentiles, respectively. n.s., no significance. ***$p < 0.001$.

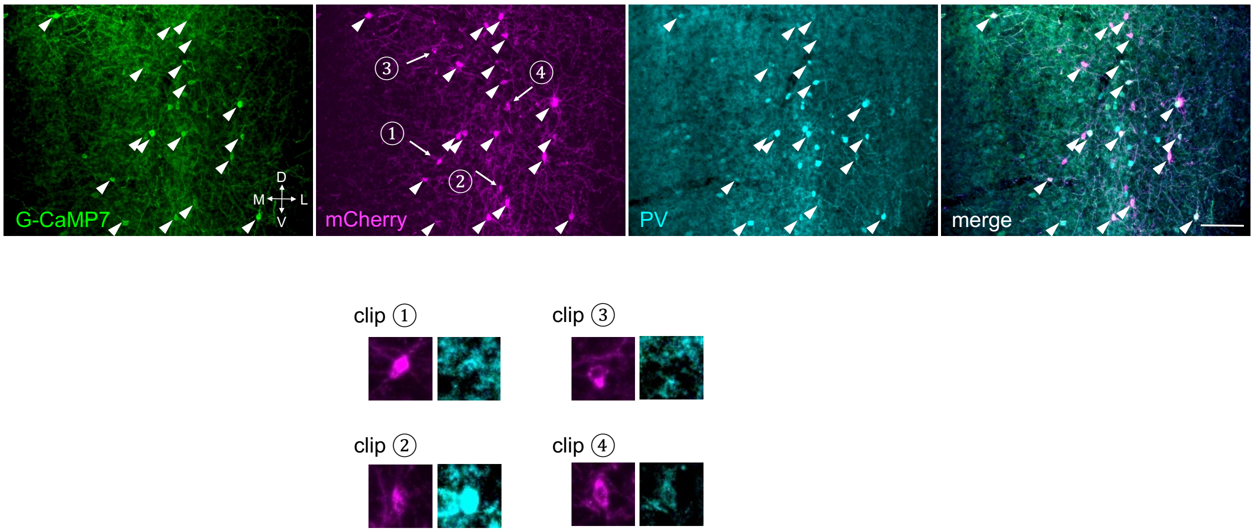

Fig. S2. Expression of G-CaMP7 and mCherry, and PV immunostaining after calcium imaging, related to Fig. 2. Representative images of G-CaMP7 (green), mCherry (magenta), and PV immunostaining (cyan) in the PL of PV-Cre mice that previously received AAV-hSyn-DIO-G-CaMP7 and AAV-hSyn-DIO-hM3D(Gq)-mCherry. Top: White arrowheads indicate triple-labeled cells (G-CaMP7+, mCherry+, and PV+). Scale bar: 100 µm. Bottom: The clipped images indicate that all mCherry positive cells have the PV signal.

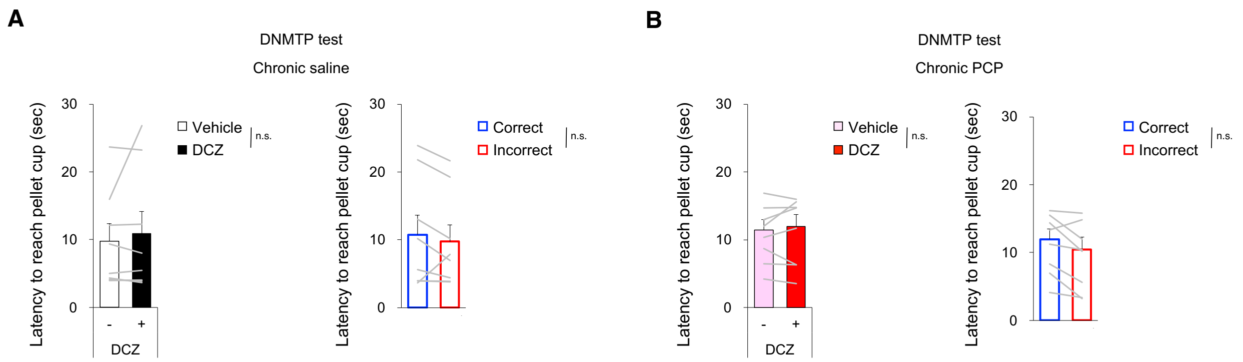

Fig. S3. The response latency did not change with DCZ or response selections, related to Fig. 2. Latency to reach pellet cup during test sessions in DNMTP task. **(A)** In chronic saline-treated PV-Cre mice, the response latency to reach pellet cup stayed the same regardless of DCZ treatment (left) or behavioral responses (right). **(B)** In chronic PCP-treated PV-Cre mice, the response latency to reach pellet cup stayed the same regardless of DCZ treatment (left) or behavioral responses (right). These results suggest that response latency is not altered by treatment conditions or response selections. Data are presented as the mean ± SEM. Each line represents each mouse. n.s., no significance.

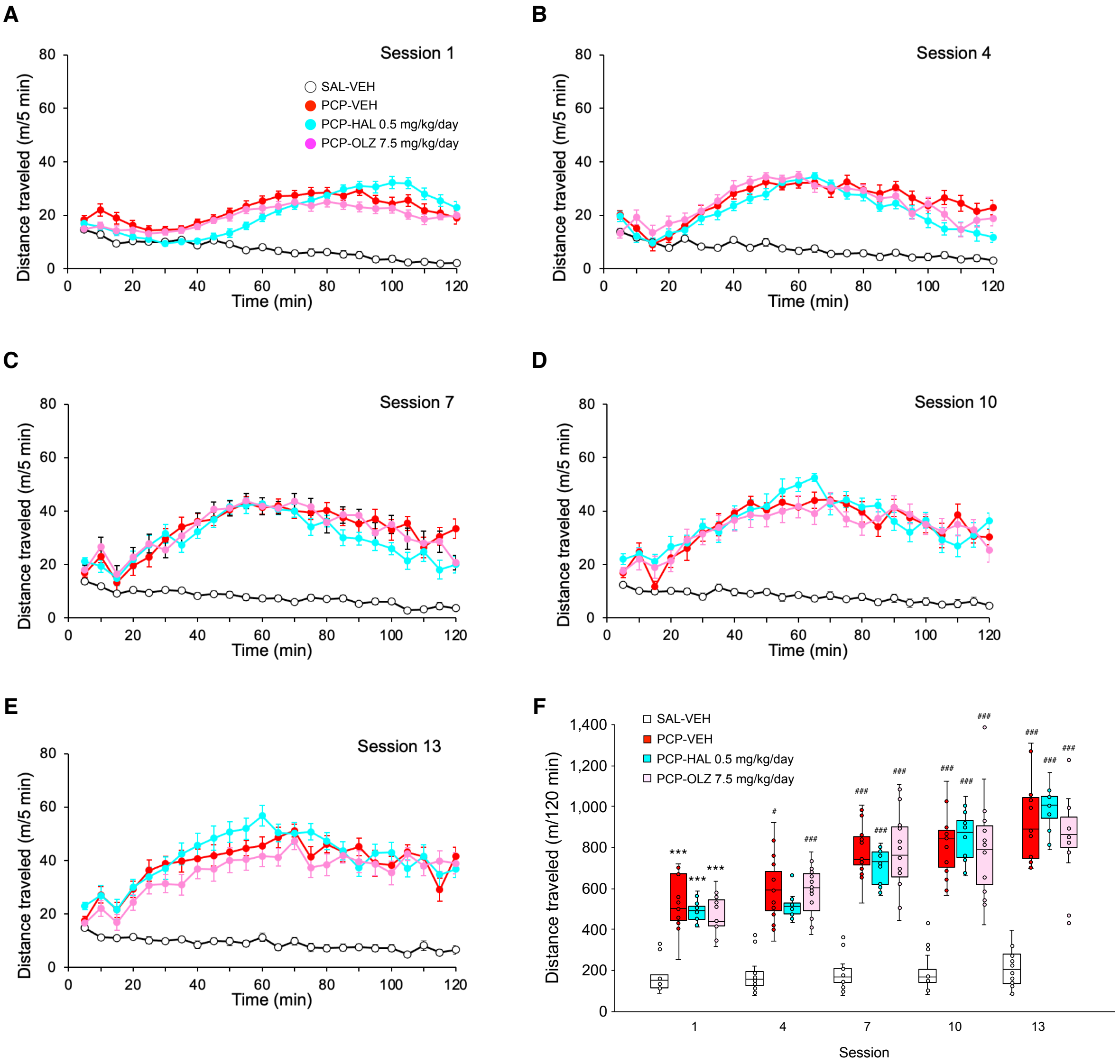

Fig. S4. Development of PCP sensitization, related to Fig. 4. (A–E) Sensitization of PCP-induced locomotor activity in mice. Mean distance traveled every 5 min. **(F)** Total ambulatory activity for 120 min in the open field. In box plots, the central mark indicates the median and the bottom and top edges of the box indicate the 25th and 75th percentiles, respectively. $^{***}p < 0.001$ vs SAL-VEH in session 1. $^{\#}p < 0.05$, $^{\#\#\#}p < 0.001$ vs session 1 in the same group.

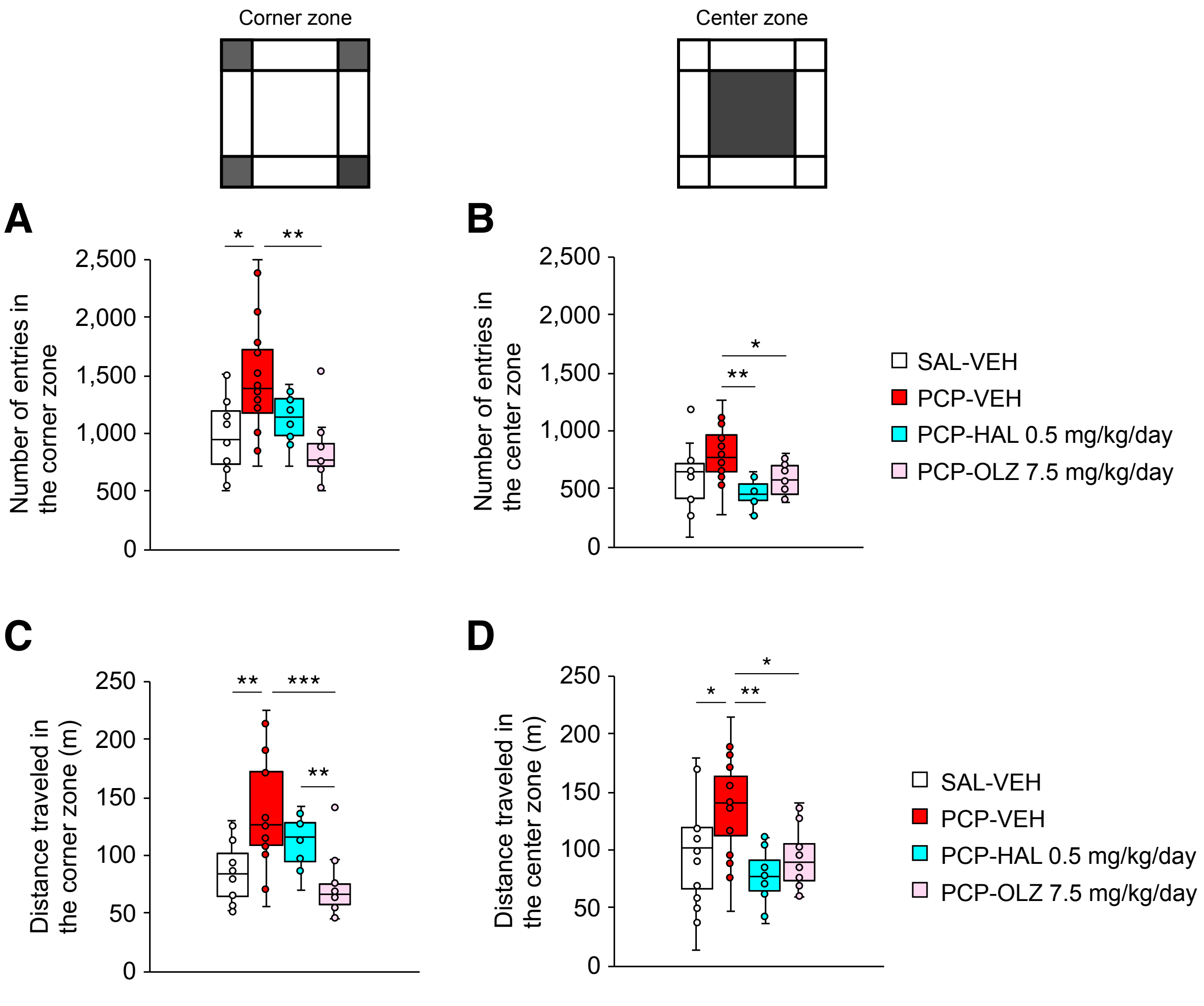

Fig. S5. Effects of acute PCP challenge on locomotor activity pattern during continuous antipsychotic treatment, related to Fig. 4. Behavioral parameters for the open field test. Number of entries into the corner **(A)** and center zone **(B)**. Total distance traveled in the corner **(C)** and center zone **(D)**. Olanzapine 7.5 mg/kg/day reversed altered behavioral patterns in chronic PCP-treated mice during open field test. In box plots, the central mark indicates the median and the bottom and top edges of the box indicate the 25th and 75th percentiles, respectively. $*p < 0.05$, $**p < 0.01$, and $***p < 0.001$.

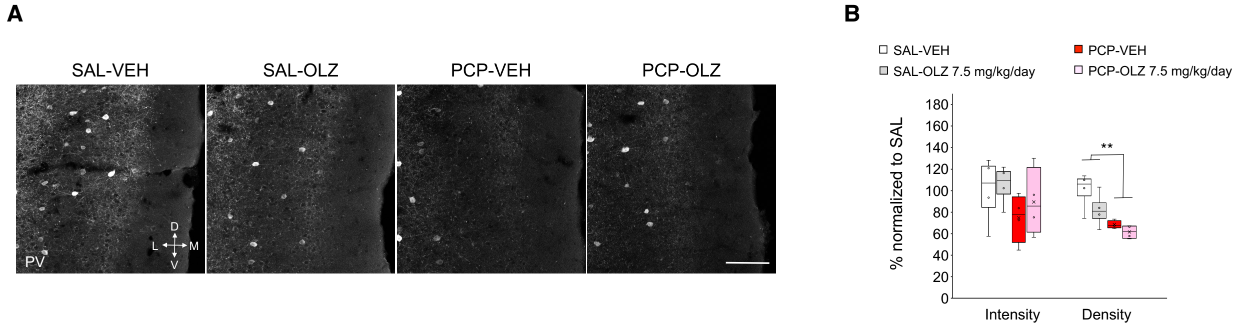

Fig. S6. Effects of chronic PCP administration and continuous olanzapine treatment on PV neuron density in the PL, related to Fig. 4. (A) Representative examples of immunohistochemical images. Scale bar: 100 µm. **(B)** Chronic PCP treatment significantly decreased the density of PV immunoreactive cells compared to chronic saline group. Olanzapine (7.5 mg/kg/day) for 2 weeks did not affect PV neuron density both in chronic saline and PCP groups. Density: quantitative analysis of PV immunoreactive cell density. Intensity: quantitative analysis of fluorescent intensity of PV immunoreactivity. In box plots, the central mark indicates the median and the bottom and top edges of the box indicate the 25th and 75th percentiles, respectively. **$p < 0.01$.

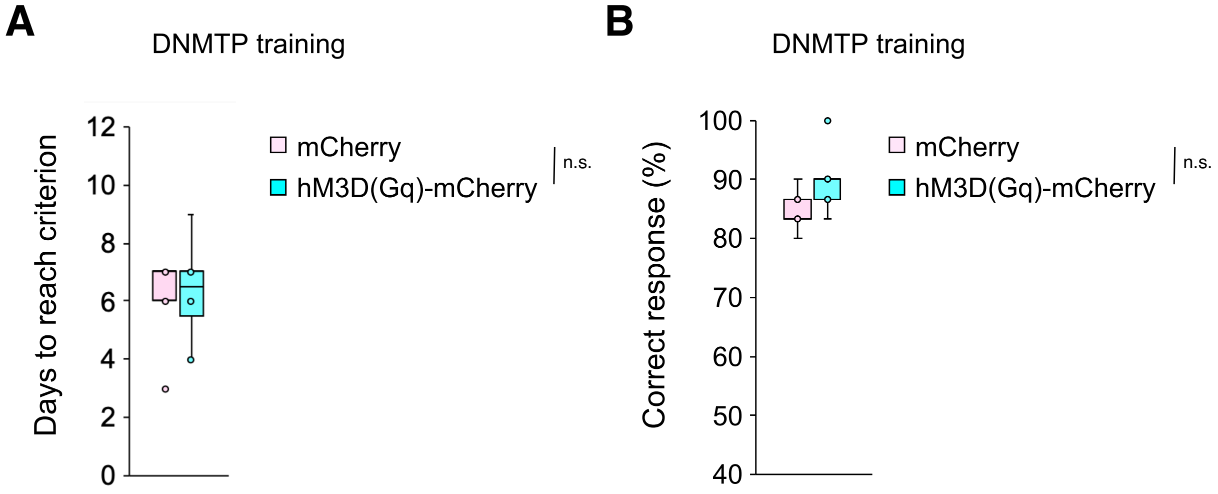

Fig. S7. Days to reach criterion and correct responses during DNMTP training phase, related to Fig. 5. Before repeated administration of PCP, no significant differences were observed in number of days taken to reach criterion **(A)** and correct responses **(B)** were observed between mCherry- and hM3D(Gq)-mCherry-expressing mice. In box plots, the central mark indicates the median and the bottom and top edges of the box indicate the 25th and 75th percentiles, respectively. n.s., no significance.

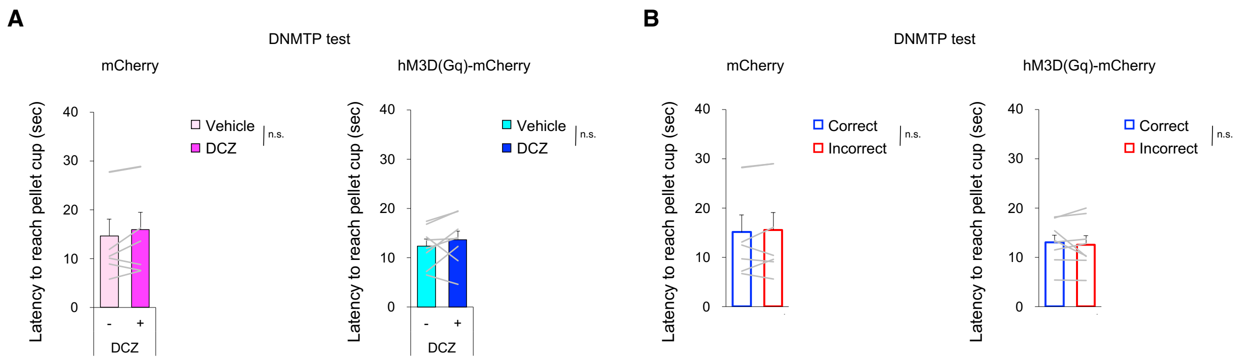

Fig. S8. The response latency did not change with DCZ or response selections, related to Fig. 5. Latency to reach pellet cup during test sessions in DNMTP task. **(A)** Both in mCherry- and hM3D(Gq)-expressing mice, no significant differences in latency to reach pellet cup were observed between vehicle and DCZ treatments. **(B)** Both in mCherry- and hM3D(Gq)-expressing mice, no significant differences in latency to reach pellet cup were observed between correct and incorrect responses. These results suggest response latency is not altered by treatment conditions or response selections. Data are presented as the mean ± SEM. Each line represents each mouse. n.s., no significance.

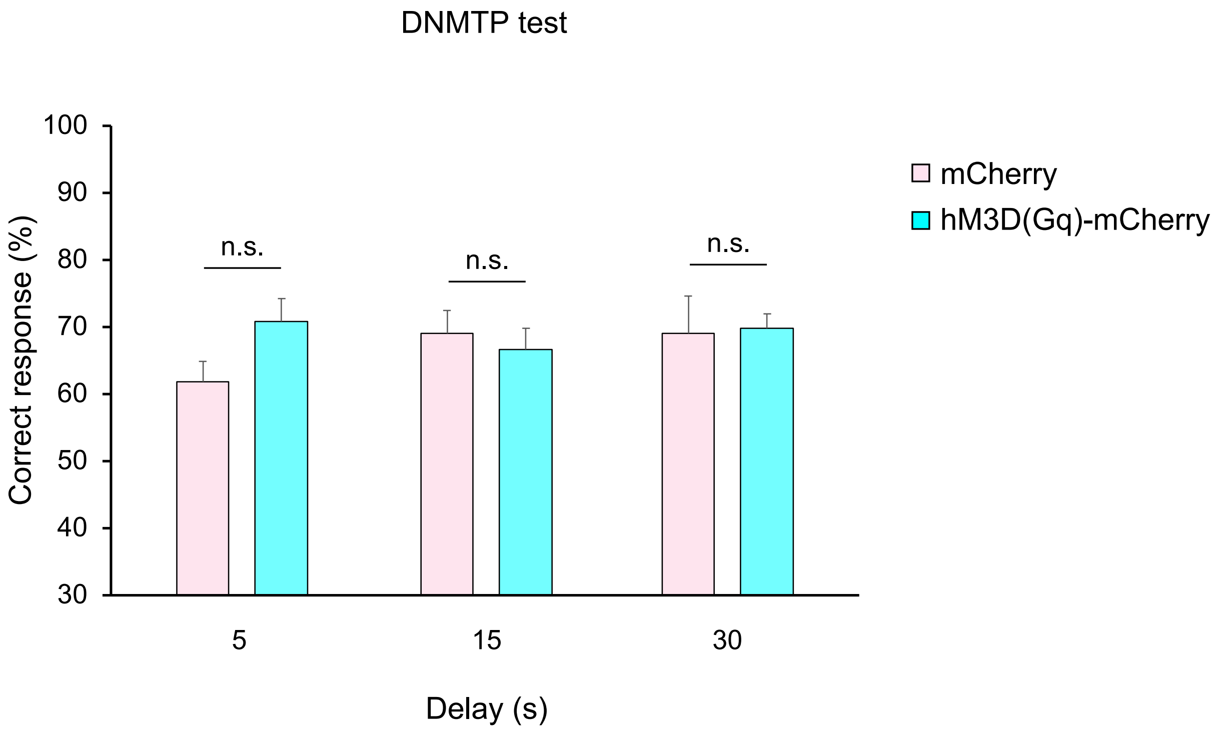

Fig. S9. In chronic PCP-treated PV-Cre mice, correct response rate did not differ between vehicle-treated mCherry and hM3D(Gq) groups in each delay time, related to Fig. 5. In the absence of DCZ, no significant differences in percent correct responses were observed between mCherry- and hM3D(Gq)-expressing mice under continuous treatment with OLZ 7.5 mg/kg/day. Data are presented as the mean ± SEM. n.s., no significance.

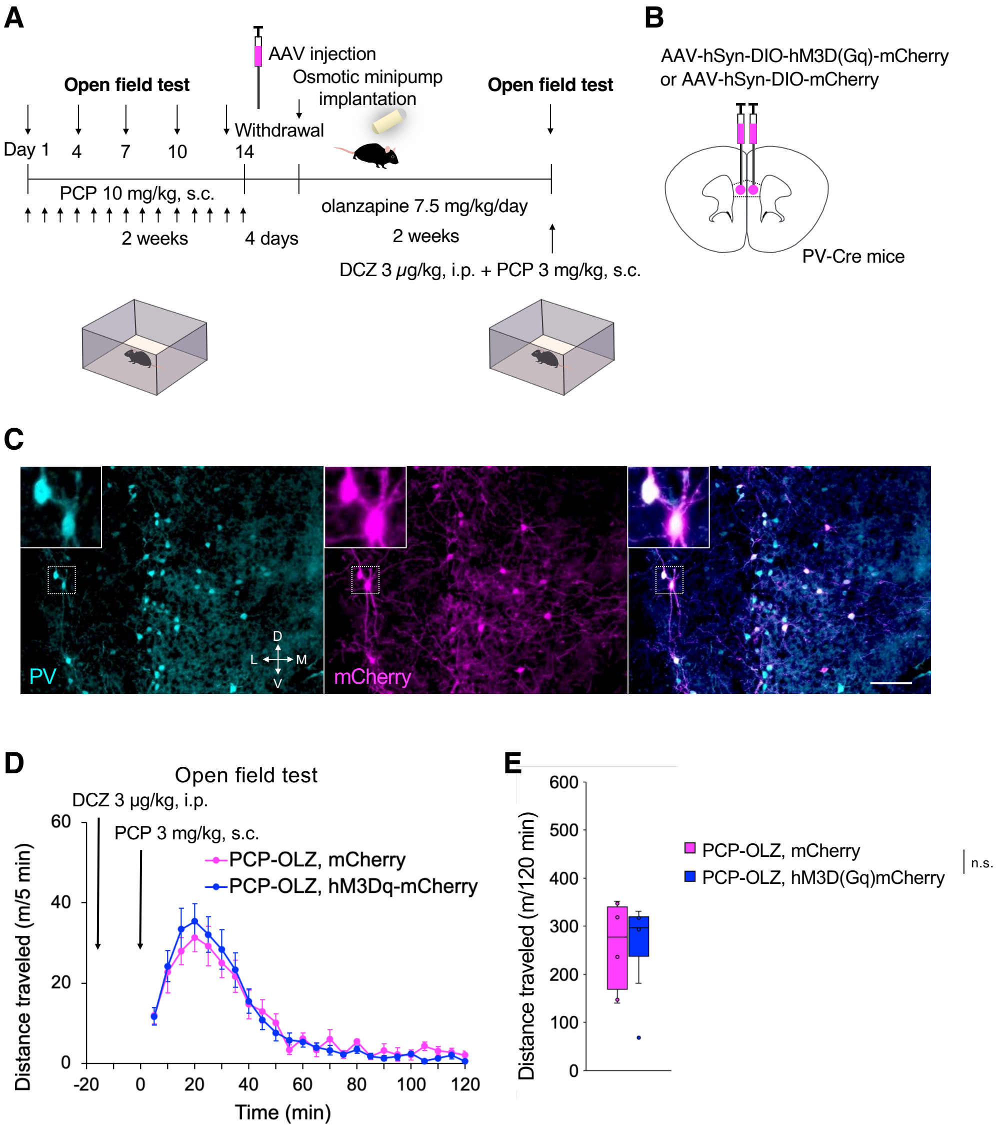

Fig. S10. Effects of chemogenetic prefrontal PV activation on acute PCP challenge with continuous olanzapine treatment, related to Fig. 5. (A) Schematic timelines. **(B)** Schema of bilateral viral delivery of AAV-hSyn-DIO-mCherry or AAV-hSyn-DIO-hM3D(Gq)-mCherry to PL. **(C)** Representative images of PV immunostaining (cyan) and viral expression (magenta) in the PL. Scale bar: 100 µm. **(D)** Mean distance traveled every 5 min for both mCherry- and hM3D(Gq)-expressing mice administered continuous olanzapine treatment. **(E)** Total distance traveled in 120 min.

In box plots, the central mark indicates the median and the bottom and top edges of the box indicate the 25th and 75th percentiles, respectively. n.s., no significance.

Supplementary Tables

Table S2. MRM transitions and analytical conditions

**Analytes**	**MRM transitions (m/z)**		**Analytical conditions**									
	Q1	Q3	DP	CE	EP	CXP	CUR	IS	TEM	GS1	GS2	CAD
S-raclopride	347.1	112.1	70	35	10	8	10	4500	700	40	70	9
metoprolol	268.4	116.1	81	31								

*DP declustering potential; CE collision energy; EP entrance potential; CXP cell exit potential; CUR curtain gas; IS ion source voltage; TEM temperature of ion source; GS1 source gas 1; GS2 source gas 2; CAD collision activated dissociation

Table S3. Effect of S-raclopride dose on mouse striatal/cerebellar S-raclopride concentration ratio

Dose	Sampling time	Ratio
3 μg/kg, i.v. (n = 5)	30 min	7.88 ± 0.66
10 μg/kg, i.v. (n = 3)	30 min	6.79 ± 0.65
30 μg/kg, i.v. (n = 3)	30 min	3.79 ± 0.43

Table S4. Effect of sampling time on mouse striatal/cerebellar S-raclopride concentration ratio

Dose	Sampling time	Ratio
3 µg/kg, i.v. (n = 4)	5 min	2.69 ± 0.09
3 µg/kg, i.v. (n = 3)	15 min	6.98 ± 0.95
3 µg/kg, i.v. (n = 5)	30 min	7.88 ± 0.66

Table S5. Dopamine D2 receptor occupancy at different doses of antipsychotics

Drug	Doses	Sampling time	D2 receptor occupancy (%)
Haloperidol	0.25 mg/kg/day	Day 3	79.6 ± 2.95
		Day 13	48.8 ± 3.18
	0.5 mg/kg/day	Day 3	80.6 ± 2.97
		Day 13	63.5 ± 3.55
	0.75 mg/kg/day	Day 3	90.7 ± 1.13
		Day 13	78.3 ± 1.19
Olanzapine	3.0 mg/kg/day	Day 3	48.1 ± 6.49
		Day 13	48.4 ± 7.39
	7.5 mg/kg/day	Day 3	80.9 ± 3.70
		Day 13	70.8 ± 2.80
		Day 28	67.2 ± 2.51
	10 mg/kg/day	Day 3	79.9 ± 1.44
		Day 13	73.4 ± 2.78
